# Changes in Health Care Costs, Survival, and Time Toxicity in the Era of Immunotherapy and Targeted Systemic Therapy for Melanoma

**DOI:** 10.1001/jamadermatol.2023.3179

**Published:** 2023-09-06

**Authors:** Sarah B. Bateni, Paul Nguyen, Antoine Eskander, Soo Jin Seung, Nicole Mittmann, Matthew Jalink, Arjun Gupta, Kelvin K. W. Chan, Nicole J. Look Hong, Timothy P. Hanna

**Affiliations:** 1 Odette Cancer Centre, Sunnybrook Health Sciences Centre, Toronto, Ontario, Canada; 2 Division of General Surgery, University of Toronto, Toronto, Ontario, Canada; 3 Division of Surgical Oncology, Department of Surgery, University of Alabama at Birmingham; 4 ICES at Queen’s University, Kingston, Ontario, Canada; 5 Institute of Health Policy, Management, and Evaluation, University of Toronto, Toronto, Ontario, Canada; 6 Department of Otolaryngology–Head and Neck Surgery, University of Toronto, Toronto, Ontario, Canada; 7 Health Outcomes and PharmacoEconomics (HOPE) Research Centre, Sunnybrook Research Institute, Toronto, Ontario, Canada; 8 Sunnybrook Research Institute, Sunnybrook Health Sciences Centre, Toronto, Ontario, Canada; 9 Division of Cancer Care and Epidemiology, Queen’s Cancer Research Institute, Kingston, Ontario, Canada; 10 Department of Public Health Sciences, Queen’s University, Kingston, Ontario, Canada; 11 Division of Hematology, Oncology, & Transplantation, University of Minnesota, Minneapolis; 12 Department of Oncology, Queen’s University, Kingston, Ontario, Canada

## Abstract

**Importance:**

Melanoma treatment has evolved during the past decade with the adoption of adjuvant and palliative immunotherapy and targeted therapies, with an unclear impact on health care costs and outcomes in routine practice.

**Objective:**

To examine changes in health care costs, overall survival (OS), and time toxicity associated with primary treatment of melanoma.

**Design, Setting, and Participants:**

This cohort study assessed a longitudinal, propensity score (PS)–matched, retrospective cohort of residents of Ontario, Canada, aged 20 years or older with stages II to IV cutaneous melanoma identified from the Ontario Cancer Registry from January 1, 2018, to March 31, 2019. A historical comparison cohort was identified from a population-based sample of invasive melanoma cases diagnosed from the Ontario Cancer Registry from January 1, 2007, to December 31, 2012. Data analysis was performed from October 17, 2022, to March 13, 2023.

**Exposures:**

Era of melanoma diagnosis (2007-2012 vs 2018-2019).

**Main Outcomes and Measures:**

The primary outcomes were mean per-capita health care and systemic therapy costs (Canadian dollars) during the first year after melanoma diagnosis, time toxicity (days with physical health care contact) within 1 year of initial treatment, and OS. Standardized differences were used to compare costs and time toxicity. Kaplan-Meier methods and Cox proportional hazards regression were used to compare OS among PS-matched cohorts.

**Results:**

A PS-matched cohort of 731 patients (mean [SD] age, 67.9 [14.8] years; 437 [59.8%] male) with melanoma from 2018 to 2019 and 731 patients (mean [SD] age, 67.9 [14.4] years; 440 [60.2%] male) from 2007 to 2012 were evaluated. The 2018 to 2019 patients had greater mean (SD) health care (including systemic therapy) costs compared with the 2007 to 2012 patients ($47 886 [$55 176] vs $33 347 [$31 576]), specifically for stage III ($67 108 [$57 226] vs $46 511 [$30 622]) and stage IV disease ($117 450 [$79 272] vs $47 739 [$37 652]). Mean (SD) systemic therapy costs were greater among 2018 to 2019 patients: stage II ($40 823 [$40 621] vs $10 309 [$12 176]), III ($55 699 [$41 181] vs $9764 [$12 771]), and IV disease ($79 358 [$50 442] vs $9318 [$14 986]). Overall survival was greater for the 2018 to 2019 cohort compared with the 2007 to 2012 cohort (3-year OS: 74.2% [95% CI, 70.8%-77.2%] vs 65.8% [95% CI, 62.2%-69.1%], hazard ratio, 0.72 [95% CI, 0.61-0.85]; *P* < .001). Time toxicity was similar between eras. Patients with stage IV disease spent more than 1 day per week (>52 days) with physical contact with the health care system by 2018 to 2019 (mean [SD], 58.7 [43.8] vs 44.2 [26.5] days; standardized difference, 0.40; *P* = .20).

**Conclusions and Relevance:**

This cohort study found greater health care costs in the treatment of stages II to IV melanoma and substantial time toxicity for patients with stage IV disease, with improvements in OS associated with the adoption of immunotherapy and targeted therapies. These health system–wide data highlight the trade-off with adoption of new therapies, for which there is a greater economic burden to the health care system and time burden to patients but an associated improvement in survival.

## Key Points

**Question:**

How have health care costs, survival, and time toxicity changed after the adoption of adjuvant and palliative immunotherapies and targeted therapies for melanoma?

**Findings:**

This cohort study, which evaluated matched cohorts of 731 patients with melanoma, found a substantial increase in systemic therapy costs in 2018 to 2019 compared with 2007 to 2012. Survival improved for all stages in 2018 to 2019 compared with 2007 to 2012, and time toxicity was similar between eras.

**Meaning:**

These data highlight the trade-off with new effective therapies, for which there are greater health care costs and time toxicity but an associated improvement in patient survival.

## Teaser

This cohort study compares costs, survival, and time toxicity among patients with melanoma in 2007 to 2012 vs 2018 to 2019.

## Introduction

Health care costs for cancer treatment are escalating, with approximately $173 billion in the US and more than $7 billion in Canada during 2020 alone.^1,2^ Because melanoma is the eighth most common cancer in Canada and the fifth most common in the US, it represents a source of significant health care expenditure.^3,4,5^ In recent years, checkpoint inhibitors (programmed death 1 [PD-1] and cytotoxic T-lymphocyte–associated protein 4 [CTLA-4] inhibitors) and targeted therapies (BRAF and MEK inhibitors) have become the standard of care for patients with locally advanced and metastatic melanoma.^6,7^ Ipilimumab, a CTLA-4 inhibitor, was first approved in Canada in 2012 for the treatment of advanced melanoma based on randomized clinical trials showing improvement in overall survival (OS).^8,9^ Approvals for PD-1 inhibitors (nivolumab and pembrolizumab) and BRAF/MEK inhibitors (dabrafenib and trametinib) followed for the treatment of metastatic disease and as adjuvant therapy in stage III disease, with improved disease-free survival and OS.^10,11,12,13^ However, these novel therapies are associated with high treatment costs, with recommended treatment duration of 1 to 2 years or longer.^6,14,15,16,17^

Surgical treatment for melanoma has also evolved in recent years, including the omission of completion lymph node dissections (CLNDs) for patients with sentinel lymph node–positive disease. Randomized clinical trials showed no difference in melanoma-specific survival and OS among patients with cutaneous melanoma randomized to receive either CLND or observation.^18,19,20^ As such, consensus guidelines no longer recommend CLND in all patients with melanoma with sentinel lymph node–positive disease.^6,21^ However, follow-up continues to involve oncologist or specialist visits and dermatologic follow-up and monitoring.

Novel cancer treatments are often accompanied by burdensome health care encounters, which can eat into the increased survival associated with that treatment. This concept is now known as *time toxicity* in reference to the amount of time spent in physical health care system contact, such as outpatient visits for bloodwork, imaging, procedures, and consultation; emergency department visits; and facility stays.^22^ Evaluating these time burdens associated with cancer care is critical to fully understand the patient and care partner experience and contextualize possible survival gains associated with new melanoma treatments.^23^

The purpose of this study was to describe the changes in health care costs, time toxicity, and survival associated with the initial treatment of melanoma using population-level data, specifically administrative data from Ontario, Canada. A value-based approach evaluates changes across multiple health outcomes achieved per dollar spent that are important to patients and care partners.^24^ We applied this method to outcomes of time toxicity and survival relative to health care costs. Because we were measuring the impact of numerous simultaneous changes in practice, a cost-effectiveness analysis was not appropriate. However, a patient-centered, value-based approach provides a means to measure the overall impact of these changes on cost and across multiple outcomes relevant to payers and patients. We hypothesized there would be a significant increase in health care utilization and, hence, in time toxicity and systemic therapy costs for patients with advanced melanoma, particularly those with stages III and IV disease, because of the use of checkpoint inhibitors and targeted systemic therapies and a decrease in frequency of completion lymphadenectomy but with improved OS.

## Methods

### Study Population and Design

We performed a retrospective cohort study of adult patients diagnosed with invasive melanoma in Ontario, Canada, from administrative data collected by the Ministry of Health. During this study period, race-based data were not available because of government privacy legislation. The primary exposure was treatment era before the COVID-19 pandemic; we compared a distinct era during which new targeted and immune-based therapies had been adopted for melanoma (2018-2019) with an era before this adoption (2007-2012). The intervening time (2013-2017) represented a transition phase with a mixture of old and new practices, challenging the interpretation of a cost-consequence study.

Ontario is the largest Canadian province, with a population of 15.5 million, and provides single-payer, universal health care coverage for adult residents through the Ministry of Health.^25^ More than 99.9% of the Ontario population are considered eligible for the Ontario Health Insurance Plan. Costing was performed from the perspective of costs to the provincial government. We identified patients aged 20 years or older who were diagnosed with cutaneous melanoma based on *International Classification of Diseases for Oncology, Third Edition* histology (872-878, 8790) and site (C44) codes from the Ontario Cancer Registry from January 1, 2018, to March 31, 2019. During this period, adjuvant and palliative targeted and immune-based therapies were approved. The American Joint Committee on Cancer (AJCC) 7th and 8th edition staging was used. Patients with missing stage information were excluded. Stage I was excluded because increased overdiagnosis of low-risk melanoma could result in inflated survival differences between eras.^26^ A historical comparison cohort was identified from a population-based sample of invasive melanoma cases diagnosed from the Ontario Cancer Registry from January 1, 2007, to December 31, 2012. This interval was used because it preceded Health Canada’s approval of checkpoint inhibitors and targeted therapies for melanoma. This study cohort and relevant findings were previously published.^27^ The current study was approved by the Queen’s University Health Sciences and Affiliated Teaching Hospitals Research Ethics Board and followed the Strengthening the Reporting of Observational Studies in Epidemiology (STROBE) and Reporting of Studies Conducted Using Observational Routinely-Collected Data (RECORD) reporting guidelines. A waiver of informed consent was granted based on the Personal Health Information Protection Act, Section 44(1). Flow diagrams for the current (2018-2019) and historical cohort (2007-2012) are described in eFigures 1 to 3 in Supplement 1.

### Data Sources and Linkage

Data were obtained from administrative data sets housed at ICES, an independent, nonprofit research institute funded by an annual grant from the Ontario Government. Cancer-specific data were abstracted from the Ontario Cancer Registry, a population-based tumor registry administered by Ontario Health. Health care utilization data were abstracted from multiple administrative databases described in the eAppendix in Supplement 1. These data sets were linked using unique encoded identifiers and analyzed at ICES.

### Covariates

Socioeconomic status was based on community-specific or neighborhood household income quintiles. Place of residence was defined by the 14 Local Health Integration Networks, which are past geographic health care partitions in Ontario. Rurality of residence was classified using the 2008 Rurality Index for Ontario (RIO). Higher scores represent a greater degree of rurality categorized as urban (RIO < 10), suburban (RIO = 10-39), or rural (RIO ≥ 40).^28^ Comorbidities were measured by using the Elixhauser comorbidity index derived from hospital records with a 5-year lookback from melanoma diagnosis.^29^

### Outcomes

Systemic therapy was identified from the Activity Level Reporting, New Drug Funding Program, and Ontario Drug Benefit databases. Individuals who received a minimum of 1 dose of either oral or intravenous systemic treatment were counted as having received systemic therapy. Radiotherapy and its intent were based on the Activity Level Reporting database. Melanoma-specific primary and nodal operations were identified from physician billing claims data. Palliative intent or metastasis operations were based on surgical intervention codes from the Discharge Abstract Database (brain, lung or liver tumors, or spinal cord compression).

Health care costs were estimated using an established costing algorithm at ICES in which person-level costs are allocated for the various health care utilizations over time.^30^ Person-level direct pharmaceutical costs for publicly funded systemic therapy administered in the Activity Level Reporting, New Drug Funding Program, and Ontario Drug Benefit databases were estimated based on patient-level utilization with a well-described algorithm developed at ICES. Although private drug plans cover limited oral drug costs for some patients younger than 65 years (eAppendix in Supplement 1), for generalizability and interpretability, costs of approved oral drugs were considered to be borne completely by the public payer; costs incurred by private insurance and privately funded clinical trials were otherwise not included.^31,32^ Oral and intravenous drug costs were included (eg, BRAF/MEK inhibitors, immunotherapy, and chemotherapy). Costs were adjusted for inflation to 2019 Canadian dollars using health care components of the Consumer Price Index. Health care costs were limited to the first year after diagnosis to ensure that stage-specific health care utilization reflected primary treatment and to prevent the influence of health care system disruptions from the COVID-19 pandemic lockdown and melanoma recurrence on results.

Overall survival was measured from the date of melanoma diagnosis to death or last follow-up. Vital status data were censored 3.5 years from diagnosis, with the latest dates of follow-up being June 30, 2015, and September 30, 2022, for the 2007 to 2012 and 2018 to 2019 cohorts, respectively. Survival was measured for a longer period than cost given the increasing absolute benefit of initial treatment over time observed in trials.^8,13^

Time toxicity was defined as in-person days with health care–related visits for any reason.^22^ This composite measure included institution-based visits (eg, hospitalizations, day operations, emergency department–only visits, and long-term care stays) and outpatient care visits (eg, cancer clinics or ambulatory interventions, primary care, and specialist office visits). We reported composite time-toxic days in the 1-year period after the first day of treatment of melanoma-specific systemic therapy, radiotherapy, and/or surgery. Home care services, virtual or telephone consultations, and physician home visits were excluded because these services did not involve time away from home. We included these encounters in a sensitivity analysis.

### Statistical Analysis

Data analysis was performed from October 17, 2022, to March 13, 2023. Propensity scores (PSs) were estimated using a logistic regression model with demographic covariates, the Elixhauser comorbidity index score, and melanoma characteristics. The PS was used to balance differences in patient characteristics between eras when comparing costs and outcomes regarding different treatment indications. Patients with melanoma diagnosed from 2007 to 2012 were matched 1:1 to patients with melanoma diagnosed from 2018 to 2019 using a greedy algorithm with calipers of 0.2 of the SD of the logit of the PS.^33^ The distributions of the PS matched characteristics between cohorts were evaluated using standardized differences, with a value of 0.10 or less suggesting adequate balance.

Mean per-person costs for health care utilization and systemic therapy were estimated with the denominators including only those who received the specific services. Differences in mean cost and time toxicity for the health care services were assessed with standardized differences. Overall survival was described using the Kaplan-Meier method, with differences between groups compared with the log-rank test and Cox proportional hazards regression. The thresholds for detecting effect size and statistical significance in all other analyses were a standardized difference of 0.20 or greater and a 2-sided *P* < .05, respectively. Data analyses were performed using SAS software, version 9.4 (SAS Institute Inc).

## Results

From 2018 to 2019, 786 patients with stages II to IV melanoma (mean [SD] age, 67.7 [14.6] years; 308 [39.2%] women and 478 [60.8%] men) were identified, and 2346 patients with melanoma (mean [SD] age, 66.3 [16.1] years; 928 [39.6%] women and 1418 [60.4%] men) were included from 2007 to 2012 (eFigures 1-3 in Supplement 1). Table 1 and eTables 1 to 3 in Supplement 1 present patient demographic, disease, and clinical characteristics of the unmatched and matched cohorts. After PS matching (731 patients per era), there were few differences (standardized difference, <0.10) in the effect sizes for demographic and disease characteristics between the 2018 to 2019 cohort (mean [SD] age, 67.9 [14.8] years; 294 [40.2%] female and 437 [59.8%] male) and 2007 to 2012 cohort (mean [SD] age, 67.9 [14.4] years; 291 [39.8%] female and 440 [60.2%] male) but notable differences in CLND (88 [12.0%] vs 226 [30.9%]; standardized difference, 0.47), wide local excision (354 [48.4%] vs 442 [60.5%]; standardized difference, 0.24), flap surgery (204 [27.9%] vs 130 [17.8%]; standardized difference, 0.24), and systemic therapy (248 [33.9%] vs 161 [22.0%]; standardized difference, 0.27).

**Table 1.** Patient Characteristics for the Unmatched and Matched Cohorts^a^

Characteristic	Unmatched		Standardized difference^b^	Matched		Standardized difference^b^
	2007-2012 (n = 2346)	2018-2019 (n = 786)		2007-2012 (n = 731)	2018-2019 (n = 731)	
Age, mean (SD), y	66.3 (16.1)	67.7 (14.6)	0.09	67.9 (14.4)	67.9 (14.8)	0.00
Age, y (categorized)						
20-39	157 (6.7)	33 (4.2)	0.11	34 (4.7)	32 (4.4)	0.01
40-49	231 (9.9)	47 (6.0)	0.14	39 (5.3)	46 (6.3)	0.04
50-59	357 (15.2)	134 (17.1)	0.05	129 (17.7)	120 (16.4)	0.03
60-69	489 (20.8)	206 (26.2)	0.13	172 (23.5)	185 (25.3)	0.04
70-79	553 (23.6)	183 (23.3)	0.01	180 (24.6)	172 (23.5)	0.03
≥80	559 (23.8)	183 (23.3)	0.01	177 (24.2)	176 (24.1)	0.00
Sex						
Female	928 (39.6)	308 (39.2)	0.01	291 (39.8)	294 (40.2)	0.01
Male	1418 (60.4)	478 (60.8)	0.01	440 (60.2)	437 (59.8)	0.01
Income quintile						
1 (Lowest)	403 (17.2)	136 (17.3)	0.00	119 (16.3)	123 (16.8)	0.01
2	447 (19.1)	145 (18.5)	0.02	151 (20.7)	137 (18.7)	0.05
3	453 (19.3)	164 (20.9)	0.04	146 (20.0)	151 (20.7)	0.02
4	498 (21.2)	156 (19.9)	0.03	134 (18.3)	149 (20.4)	0.05
5 (Highest)	545 (23.2)	185 (23.5)	0.01	181 (24.8)	171 (23.4)	0.03
Place of residence						
Erie St Clair	146 (6.2)	52 (6.6)	0.02	47 (6.4)	50 (6.8)	0.02
South West	230 (9.8)	78 (9.9)	0.00	72 (9.9)	73 (10.0)	0.00
Waterloo Wellington	159 (6.8)	65 (8.3)	0.06	65 (8.9)	59 (8.1)	0.03
HNHB	365 (15.6)	90 (11.5)	0.12	98 (13.4)	88 (12.0)	0.04
Central West	77 (3.3)	21 (2.7)	0.04	24 (3.3)	20 (2.7)	0.03
Mississauga Halton	156 (6.7)	32 (4.1)	0.11	32 (4.4)	31 (4.2)	0.01
Toronto Central	163 (7.0)	62 (7.9)	0.04	50 (6.8)	58 (7.9)	0.04
Central	205 (8.7)	75 (9.5)	0.03	75 (10.3)	70 (9.6)	0.02
Central East	290 (12.4)	87 (11.1)	0.04	82 (11.2)	86 (11.8)	0.02
South East	149 (6.4)	54 (6.9)	0.02	52 (7.1)	51 (7.0)	0.01
Champlain	155 (6.6)	91 (11.6)	0.17	58 (7.9)	71 (9.7)	0.06
North Simcoe Muskoka	109 (4.7)	36 (4.6)	0.00	39 (5.3)	36 (4.9)	0.02
North East or North West	142 (6.1)	43 (5.5)	0.02	37 (5.1)	38 (5.2)	0.01
Urban or rural residence						
Urban	1453 (61.9)	479 (60.9)	0.02	445 (60.9)	442 (60.5)	0.01
Suburban	650 (27.7)	222 (28.2)	0.01	211 (28.9)	210 (28.7)	0.00
Rural	243 (10.4)	85 (10.8)	0.01	75 (10.3)	79 (10.8)	0.02
Elixhauser comorbidity index, mean (SD)	0.64 (1.30)	0.63 (1.32)	0.01	0.67 (1.26)	0.63 (1.32)	0.03
Elixhauser comorbidity index (categorized)						
0-1	1968 (83.9)	661 (84.1)	0.01	601 (82.2)	617 (84.4)	0.06
2-3	259 (11.0)	91 (11.6)	0.02	96 (13.1)	82 (11.2)	0.06
≥4	119 (5.1)	34 (4.3)	0.04	34 (4.7)	32 (4.4)	0.01
Histology (categorized)						
Melanoma, NOS	544 (23.2)	309 (39.3)	0.35	280 (38.3)	259 (35.4)	0.06
Nodular melanoma	832 (35.5)	259 (33.0)	0.05	236 (32.3)	256 (35.0)	0.06
Lentigo maligna or acral lentiginous melanoma	120 (5.1)	44 (5.6)	0.02	39 (5.3)	42 (5.8)	0.02
Superficial spreading melanoma	530 (22.6)	124 (15.8)	0.17	127 (17.4)	124 (17.0)	0.01
Other	320 (13.6)	50 (6.4)	0.24	49 (6.7)	50 (6.8)	0.01
Body site (categorized)						
Head or neck	511 (21.8)	141 (17.9)	0.10	146 (20.0)	137 (18.7)	0.03
Trunk	744 (31.7)	247 (31.4)	0.01	236 (32.3)	230 (31.5)	0.02
Arm or shoulder	560 (23.9)	197 (25.1)	0.03	176 (24.1)	191 (26.1)	0.05
Leg, hip, or other	531 (22.6)	201 (25.6)	0.07	173 (23.7)	173 (23.7)	0.00
Best stage						
II	1524 (65.0)	421 (53.6)	0.23	421 (57.6)	421 (57.6)	0.00
III	737 (31.4)	260 (33.1)	0.04	254 (34.8)	254 (34.8)	0.00
IV	85 (3.6)	105 (13.4)	0.35	56 (7.7)	56 (7.7)	0.00
Treatment						
Primary surgery^c^						
WLE	1403 (59.8)	363 (46.2)	0.28	442 (60.5)	354 (48.4)	0.24
Amputation	69 (2.9)	17 (2.2)	0.05	29 (4.0)	17 (2.3)	0.09
Graft	172 (7.3)	68 (8.7)	0.05	56 (7.7)	66 (9.0)	0.05
Flap	475 (20.3)	210 (26.7)	0.15	130 (17.8)	204 (27.9)	0.24
Other excisions	130 (5.5)	43 (5.5)	0.00	33 (4.5)	40 (5.5)	0.04
None	97 (4.1)	85 (10.8)	0.26	41 (5.6)	50 (6.8)	0.05
Nodal surgery						
SLNB	1491 (63.6)	495 (63.0)	0.01	447 (61.1)	485 (66.3)	0.11
CLND	666 (28.4)	99 (12.6)	0.40	226 (30.9)	88 (12.0)	0.47
Systemic therapy^d^	473 (20.2)	293 (37.3)	0.39	161 (22.0)	248 (33.9)	0.27
Radiotherapy	237 (10.1)	98 (12.5)	0.07	90 (12.3)	79 (10.8)	0.05
Metastasis surgery^e^	9 (0.38)	20 (2.5)	0.18	7 (1.0)	14 (1.9)	0.08

Abbreviations: CLND, completion lymph node dissection; HNHB, Hamilton Niagara Haldimand Brant; NOS, not otherwise specified; SLNB, sentinel lymph node biopsy; WLE, wide local excision.

^a^ Data are presented as number (percentage) of patients unless otherwise indicated.

^b^ A standardized difference of 0.10 or less suggests a balance in effect size between groups.

^c^ If more than 1 type of primary surgery occurred, treatment is prioritized in the following order: amputation, WLE, graft, flap, and other excision.

^d^ Systemic therapy patients had a mean (SD) of 11.7 (7.9) visits or a median (IQR) of 10 (5-18) visits for injection and/or oral systemic therapy.

^e^ Metastasis surgery includes operations for spinal cord, brain, lung, and liver metastases.

Table 2 describes the mean health care utilization and systemic therapy per-person costs within the first year of diagnosis for the matched cohort (eTable 4 in Supplement 1 provides unmatched costs). Patients from the 2018 to 2019 cohort had higher mean (SD) overall health care per-person costs compared with patients in the 2007 to 2012 patients cohort ($47 886 [$55 176] vs $33 347 [$31 576]; standardized difference, 0.32), mainly contributed to by patients with stage III ($67 108 [$57 226] vs $46 511 [$30 622]; standardized difference, 0.45) and stage IV disease ($117 450 [$79 272] vs $47 739 [$37 652]; standardized difference, 1.12). Excluding systemic therapy costs, patients in the 2018 to 2019 cohort had higher mean (SD) costs compared with those in the 2007 to 2012 cohort for stage IV disease ($60 765 [$44 415] vs $44 744 [$35 865]; standardized difference, 0.40) but lower mean costs for stage III disease ($37 065 [$25 246] vs $42 821 [$27 100]; standardized difference, 0.22). Mean systemic therapy costs were significantly greater among patients in the 2018 to 2019 cohort compared with those in the 2007 to 2012 cohort (stage II disease: $40 823 [$40 621] vs $10 309 [$12 176]; standardized difference, 1.02; stage III disease: $55 699 [$41 181] vs $9764 [$12 771]; standardized difference, 1.51; and stage IV disease: $79 358 [$50 442] vs $9318 [$14 986]; standardized difference, 1.88), driven by utilization of BRAF/MEK and checkpoint inhibitor therapies (Table 2; eTables 5 and 6 in Supplement 1).

**Table 2.** Mean Health Care Utilization Per-Person Costs in Canadian Dollars for the Matched Cohorts Stratified by Stage Within the First Year After Melanoma Diagnosis

Cost description	Stage II		Stage III		Stage IV		All patients	
	2007-2012 (n = 421)	2018-2019 (n = 421)	2007-2012 (n = 254)	2018-2019 (n = 254)	2007-2012 (n = 56)	2018-2019 (n = 56)	2007-2012 (n = 731)	2018-2019 (n = 731)
Short episodes (<60 d)								
Inpatient hospitalization cost	13 540 (15 620)	13 123 (17 309)	11 959 (13 328)	13 795 (19 196)	18 234 (16 676)^a^	24 316 (31 252)^a^	13 438 (14 828)	15 068 (20 950)
Hospital outpatient clinic visit cost	2800 (2235)	3103 (2395)	4729 (3158)	4625 (2728)	4391 (3568)^a^	5388 (4077)^a^	3609 (2861)	3808 (2799)
Same-day surgery cost	2351 (1436)^a^	2942 (1422)^a^	3436 (2197)	3299 (1449)	1914 (1161)^a^	2255 (1296)^a^	2745 (1836)	3046 (1444)
ED visit cost	761 (742)	777 (743)	893 (857)	910 (830)	1101 (832)	1254 (1008)	852 (804)	886 (825)
Dialysis clinic visit cost	NR^b^	NR^b^	NR^b^	NR^b^	NA	NA	2715 (2999)	46 063 (63 477)
Oncology clinic visit cost	11 087 (14 539)	10 304 (11 038)	19 930 (15 967)	19 467 (12 347)	10 140 (11 592)^a^	18 620 (13 486)^a^	15 369 (15 630)	16 164 (12 791)
Inpatient rehabilitation cost	16 530 (7454)	22 717 (17 509)	20 733 (4034)	24 412 (15 033)	28 782 (15 628)	22 927 (6144)	23 376 (11 470)	23 087 (12 089)
Long-term episodes								
Complex continuing care cost	11 358 (11 765)	27 787 (28 649)	26 816 (35 895)	3302 (3576)	8364 (8306)	17 688 (22 259)	15 513 (22 303)	15 129 (21 480)
Long-term care cost	28 330 (16 451)	31 668 (20 994)	35 107 (8470)	30 749 (22 581)	20 012 (22 869)	18 080 (9629)	28 691 (15 823)	30 247 (20 383)
Inpatient mental health cost	NR^b^	NR^b^	NR^b^	NR^b^	NA	NA	83 644 (154 049)	63 041 (71 685)
Visits or claims								
FFS GP or FP visit cost	538 (635)	443 (571)	1078 (6281)	555 (793)	1044 (1048)	1063 (1203)	765 (3749)	535 (744)
FFS specialist visit cost	3264 (2472)^a^	3856 (3160)^a^	5431 (3055)^a^	4827 (2672)^a^	5901 (4766)^a^	7163 (5366)^a^	4220 (3118)	4447 (3347)
Non-FFS GP or FP visit cost	45 (85)^a^	26 (20)^a^	76 (110)^a^	27 (22)^a^	46 (37)^a^	30 (18)^a^	56 (21)^a^	26 (21)^a^
ED-AFA non-FFS visit cost	179 (186)	223 (153)	221 (199)	275 (230)	172 (195)	248 (226)	194 (192)	245 (196)
Non-FFS medical oncologist visit cost	677 (1459)	NA	1143 (1547)	NA	1429 (1589)	NA	945 (1529)	NA
Radiation oncologist AFA payment apportioned cost	190 (133)	NA	204 (136)	NA	251 (216)	NA	205 (149)	NA
Other non-FFS visit cost	281 (472)	463 (496)	466 (673)	997 (750)	464 (532)	1543 (1242)	379 (575)	754 (768)
Laboratory cost	329 (266)	315 (226)	296 (227)	343 (286)	345 (307)	322 (237)	319 (257)	325 (249)
Nonphysician cost	261 (465)	71 (118)	165 (335)	78 (131)	224 (385)	58 (22)	230 (426)	72 (119)
FHO or FHN physician capitation cost	234 (185)^a^	272 (166)^a^	213 (159)	226 (127)	169 (158)	197 (162)	222 (175)	251 (156)
Home care services cost	3857 (4454)	3143 (2869)	4694 (4849)^a^	3268 (3164)^a^	5449 (6390)	4436 (4764)	4408 (4869)^a^	3341 (3267)^a^
Non–anticancer-related drug cost	1569 (2015)	1273 (1947)	1743 (5650)	1628 (5661)	1973 (3932)	1944 (2407)	1661 (3793)	1446 (3638)
Systemic therapy								
Anticancer-related drug cost	10 309 (12 176)^a^	40 823 (40 621)^a^	9764 (12 771)^a^	55 699 (41 181)^a^	9318 (14 986)^a^	79 358 (50 442)^a^	9860 (12 803)^a^	56 874 (44 336)^a^
Total								
Without anticancer drug cost	22 462 (25 550)	22 575 (24 318)	42 821 (27 100)^a^	37 065 (25 246)^a^	44 744 (35 865)^a^	60 765 (44 415)^a^	31 243 (28 847)	30 535 (28 831)
With anticancer drug cost	23 491 (27 435)	27 035 (34 517)	46 511 (30 622)^a^	67 108 (57 226)^a^	47 739 (37 652)^a^	117 450 (79 272)^a^	33 347 (31 576)^a^	47 886 (55 176)^a^

Abbreviations: AFA, alternative funding arrangement; ED, emergency department; FFS, fee for service; FP, family practitioner; FHN, family health network; FHO, family health organization; GP, general practitioner; NA, not applicable; NR, not reported.

^a^ Significant effect size between groups based on a standardized difference of 0.20 or greater.

^b^ Mean (SD) costs are suppressed because number of patients can be backward calculated based on the SD costs.

**Figure 1.**
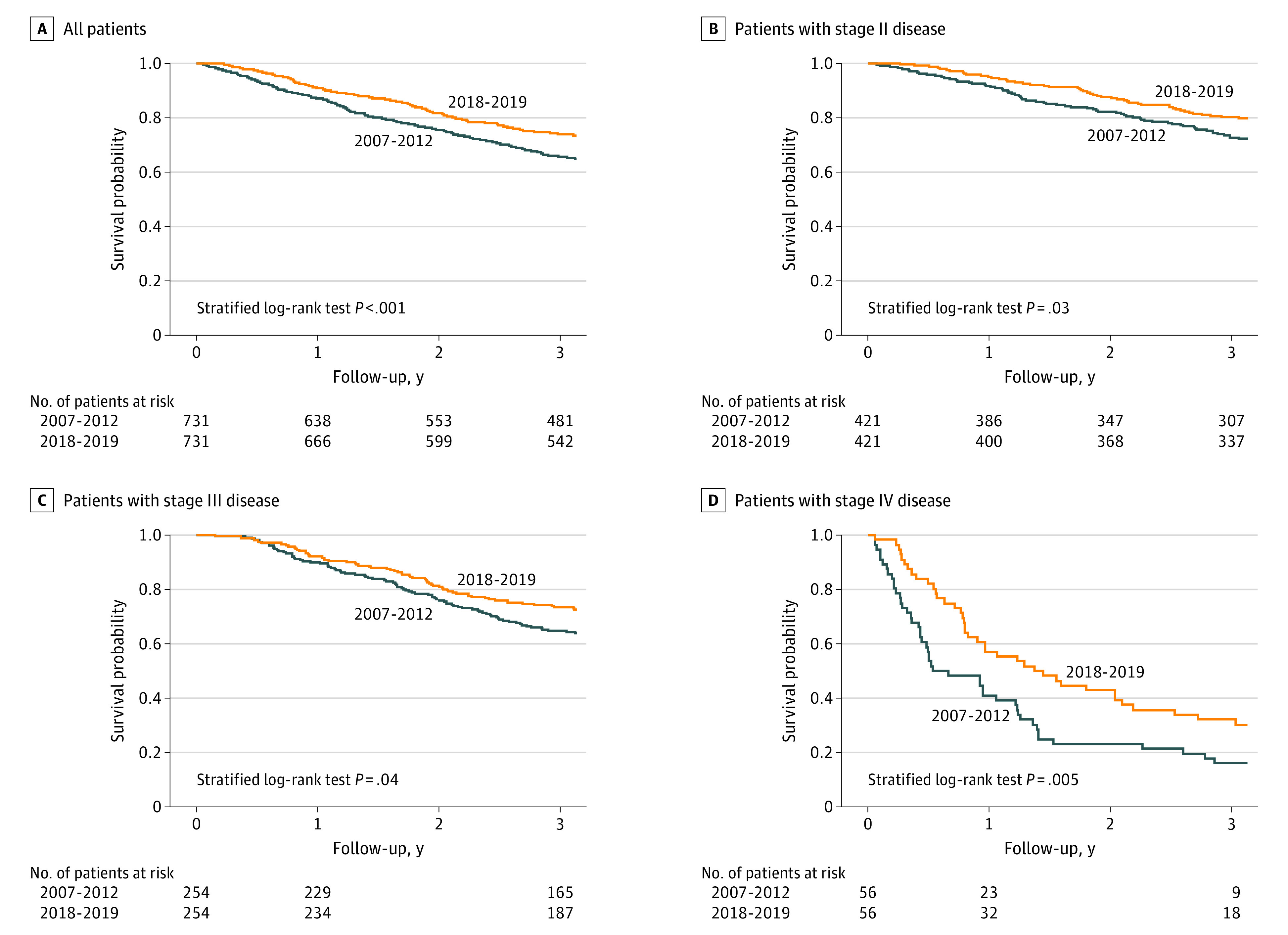
Kaplan-Meier Overall Survival Curves for the Matched Cohorts Stratified by Stage For year 2, the number of patients with stage II and stage IV disease at risk is not shown because of privacy regulations for groups of 5 or fewer patients.

As shown in Figure 1A, patients in the 2018 to 2019 cohort had higher OS compared with those in the 2007 to 2012 cohort during the 3.5-year follow-up. Three-year survival was 74.2% (95% CI, 70.8%-77.2%) for patients in the 2018 to 2019 cohort compared with 65.8% (95% CI, 62.2%-69.1%) for those in the 2007 to 2012 cohort (hazard ratio [HR], 0.72; 95% CI, 0.61-0.85; *P* < .001). Stratified by stage in Figure 1B-D, higher OS remained significant in 2018 to 2019 for patients with stage II (3-year OS: 80.1% [95% CI, 75.9%-83.6%] vs 72.9% [95% CI, 68.4%-76.9%]; HR, 0.73; 95% CI, 0.56-0.94), stage III (3-year OS: 73.6% [95% CI, 67.7%-78.6%] vs 65.0% [95% CI, 58.8%-70.5%]; HR, 0.70; 95% CI, 0.53-0.94), and stage IV (3-year OS: 32.1% [95% CI, 20.5%-44.4%] vs 16.1% [95% CI, 7.9%-26.8%]; HR, 0.57; 95% CI, 0.37-0.87) disease.

**Figure 2.**
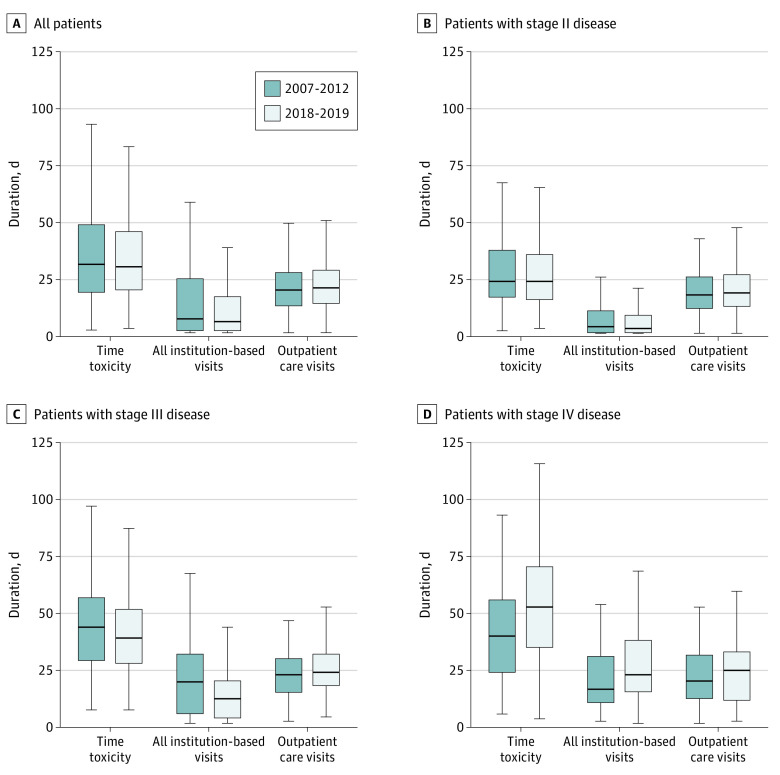
Matched Cohort of Time Toxicity and Health Utilization Within 1 Year of Treatment Initiation Among Patients With Stages II to IV Disease In the box and whisker plots, the lower and upper bounds of the boxes represent first and third quartiles, respectively, and the center lines represent medians. The lower and upper bounds of the whiskers, respectively, are calculated as first quartile – 1.5 × IQR and third quartile + 1.5 × IQR, where the IQR is third quartile – first quartile. The lower bound is set to zero if the whisker is negative. Time toxicity includes the sum of all institution-based visits and outpatient care visits. If any institution-based and outpatient care visits occured on the same day, the health care service is only counted for the institution-based visit.

Figure 2 depicts time toxicity and quantifies specific health care services within the first year after the first cancer treatment for the matched cohort. As shown in Figure 2A, few differences in time toxicity were observed between eras. Stratified by stage in Figure 2B-D, similar findings were also observed for stages II and III disease, with patients in 2018 to 2019 having few differences in overall time toxicity. In Figure 2D, the patients with stage IV disease in 2018 to 2019 had numerically greater mean (SD) time toxicity (58.7 [43.8] vs 44.2 [26.5] days; standardized difference, 0.40; *P* = .20). In a sensitivity analysis of time toxicity including home care visits and virtual visits, time toxicity substantially increased for all stages, although the proportional change in time toxicity between eras was similar (eTable 7 in Supplement 1).

## Discussion

In this population-level analysis of patients diagnosed with melanoma in Ontario, we observed a significant increase in mean health care costs for systemic therapy and health care utilization in patients with stage IV disease, with respective differences in OS and high time toxicity for stage IV disease in both eras. We observed a 6- to 9-fold increase in mean per-person costs for systemic therapy among patients with stages III and IV disease between 2007 to 2012 and 2018 to 2019. In patients with stage IV disease, there were also greater mean health care utilization per-person costs. Notably for patients, there was little association with mean health care utilization per-person costs and, relatedly, time toxicity for patients with stages II and III disease. Importantly, there was a significant improvement in OS in a PS-matched cohort of patients with stages II to IV melanoma, which likely is secondary to the greater effectiveness of checkpoint inhibitors and targeted therapies in routine practice.^10,11^ Patients with stage I disease were excluded because of the high potential for overdiagnosis. These data highlight the value trade-off with these new effective therapies in which there is a significant increase in the economic burden to the payer and continued high time burden to patients with stage IV disease, albeit with an associated improvement in OS. These systemwide trends in value of melanoma care in routine practice are relevant to other systems, both public and private.

We were able to provide a systemwide comparison of the impact of the adoption of new immunotherapies and targeted therapies for melanoma treatment. Our findings indicate higher systemic therapy costs with the adoption of immunotherapies and targeted therapies, which complements prior studies that have described the significant economic burden associated with these therapies in melanoma with respect to direct treatment costs and costs associated with therapy-related adverse events.^14,15,34,35,36,37,38,39,40^ Although there are clear clinical benefits in trials, findings from previous studies investigating the cost-effectiveness of new systemic treatments for advanced melanoma suggest that new drugs for advanced melanoma are in many cases not cost-effective.^41,42,43,44,45^ Most of these economic analyses are model based, with limited analysis of patient-level data from routine practice.^45^ Our study thus complements the existing literature by providing a population-level perspective for a whole health care system before and after adoption of multiple novel targeted and immune-based therapies for melanoma.

The significant costs of systemic therapies for the treatment of stages III and IV disease highlight the importance of early detection of melanoma, as effective early detection and referral by dermatologic specialists may decrease the burden of advanced disease and decrease costs and improve survival with melanoma.^46^ Delays in melanoma diagnosis have been shown to be associated with stage progression and worse survival.^47^ Our data suggest that by diagnosing melanoma earlier, health care costs and time toxicity may also be less, improving the health care value. Patients with stage IV disease in 2018 to 2019 experienced a mean of 58.7 time-toxic days in the year after initiating treatment; thus, patients spent almost 2 of the first 12 months, or more than 1 day per week, in a health care facility or attending outpatient appointments. These data can be used to communicate expected burdens of treatment to patients as well as guide improvement efforts, such as through care coordination.

When excluding the costs of systemic therapies, there were greater mean health care per-person costs in stage IV disease. This outcome is likely multifactorial. There was a higher mean cost associated with oncology clinic visits and inpatient hospitalizations in 2018 to 2019, which may potentially be secondary to the initiation of these newer systemic therapies requiring closer monitoring because of risks of adverse events, hospitalizations from experiencing an adverse event, and longer duration of treatment due to better survival.^15,38,39,40,48^

### Limitations

Despite the strengths of this study, we acknowledge its limitations. We limited our analysis to costs within the first year after diagnosis to examine results for initial treatment. We therefore did not include costs associated with ongoing treatment, recurrence, and surveillance beyond this point. Additionally, we did not have information with respect to the individual costs incurred to patients (indirect costs), such as the cost of transportation and lost wages, which may also have affected our time toxicity estimations. We also could not account for undisclosed discounts on drug prices negotiated by the health ministry. Our measure of time toxicity may be both an underestimate of true total time burdens faced by patients (it did not include home-based care, such as telemedicine) and an overestimate of melanoma-specific time burdens (included all health care days, not only those associated with melanoma treatment, adverse effects, or recovery). However, we expect the misclassification bias to be close to identical between the 2 treatment eras, so the differences should be proportional. Our sensitivity analysis of time toxicity, including virtual and home care visits, supports this view. We were also restricted by the administrative databases’ limitations regarding missing data with respect to disease stage. We note that the 2018 to 2019 cohort was based on cases with disease stage reported from cancer centers, effectively capturing nearly all patients treated for advanced cancers. Our matched analysis mitigated this effect. To allow inclusion of patients with AJCC 7th and 8th edition staging, stage subgroups could not be used for matching. We note that factors associated with advanced melanoma thickness and stage were reasonably balanced in the stages II to IV cohorts, mitigating against confounding due to possible changes over time in stage subgroup case mix or sentinel lymph node biopsy use according to melanoma thickness.^27^ However, we acknowledge that despite the strength of using PS matching, there may still be unmeasured confounders affecting our analysis.

## Conclusions

In this population-level, value-based cohort study, there was a significant increase in mean per-person health care costs over time, largely due to the high costs of immunotherapies and targeted systemic therapies for the treatment of advanced and metastatic melanoma. Additionally, there was continued high time toxicity in patients with stage IV melanoma. These results suggest the importance of early detection, as early-stage (stage II) melanoma was associated with much lower per-person health care utilization costs as well as improved prognosis. Furthermore, these results highlight the value trade-off of these new effective systemic therapies in which there is a greater economic burden to the health care system and time burden to patients with stage IV disease but with associated improvements in patient survival. These findings have broad implications for other cancers and other health care systems for which immunotherapies and targeted therapies are funded and are used more frequently across numerous cancer types.^49,50,51,52^

## Notes

Supplement 1. **eFigure 1.** Flow Diagram for Invasive Cutaneous Melanoma Patients and Their Pathology Records in Ontario From January 1, 2007 to December 31, 2012 **eFigure 2.** Flow Diagram for Invasive Cutaneous Melanoma Patients in Ontario From January 1, 2018 to March 31, 2019 **eFigure 3.** Flow Diagram for Propensity Score Matched Stage II-IV Melanoma Patients in Ontario From January 1, 2007 to December 31, 2012 and January 1, 2018 to March 31, 2019 **eFigure 4.** Kaplan-Meier Survival Curves for the Unmatched Cohorts Stratified by Stage **eAppendix.** Description of ICES Administrative Data Sources **eTable 1.** Patient Characteristics for Unmatched and Matched Cohorts of Stage II Patients **eTable 2.** Patient Characteristics for Unmatched and Matched Cohorts of Stage III Patients **eTable 3.** Patient Characteristics for Unmatched and Matched Cohorts of Stage IV Patients **eTable 4.** Mean Healthcare Utilization Per-Person Costs in Canadian Dollars for the Unmatched Cohorts Stratified by Stage **eTable 5.** Mean Systemic Therapy Per-Person Costs in Canadian Dollars for the Unmatched Cohorts Stratified by Stage **eTable 6.** Mean Systemic Therapy Per-Person Costs in Canadian Dollars for the Matched Cohorts Stratified by Stage **eTable 7.** Time Toxicity Sensitivity Analysis in Days Including Virtual Visits and Home Care Visits

Supplement 2.
Data Sharing Statement

## References

[1] Mariotto AB, Yabroff KR, Shao Y, Feuer EJ, Brown ML. Projections of the cost of cancer care in the United States: 2010-2020. J Natl Cancer Inst. 2011;103(2):117-128. doi:10.1093/jnci/djq495 21228314 PMC3107566

[2] de Oliveira C, Weir S, Rangrej J, . The economic burden of cancer care in Canada: a population-based cost study. CMAJ Open. 2018;6(1):E1-E10. doi:10.9778/cmajo.20170144 29301745 PMC5878959

[3] Siegel RL, Miller KD, Fuchs HE, Jemal A. Cancer statistics, 2021. CA Cancer J Clin. 2021;71(1):7-33. doi:10.3322/caac.21654 33433946

[4] Brenner DR, Weir HK, Demers AA, ; Canadian Cancer Statistics Advisory Committee. Projected estimates of cancer in Canada in 2020. CMAJ. 2020;192(9):E199-E205. doi:10.1503/cmaj.191292 32122974 PMC7055947

[5] American Cancer Society. Estimated number of new cancer cases and deaths by sex, US, 2022. Accessed February 15, 2023. https://www.cancer.org/research/cancer-facts-statistics/all-cancer-facts-figures/cancer-facts-figures-2022.html

[6] Swetter SM, Thompson JA, Albertini MR, . NCCN Guidelines® Insights: melanoma: cutaneous, version 2.2021. J Natl Compr Canc Netw. 2021;19(4):364-376. doi:10.6004/jnccn.2021.0018 33845460

[7] Petrella TM, Fletcher GG, Knight G, . Systemic adjuvant therapy for adult patients at high risk for recurrent cutaneous or mucosal melanoma: an Ontario Health (Cancer Care Ontario) clinical practice guideline. Curr Oncol. 2020;27(1):e43-e52. doi:10.3747/co.27.5933 32218667 PMC7096195

[8] Hodi FS, O’Day SJ, McDermott DF, . Improved survival with ipilimumab in patients with metastatic melanoma. N Engl J Med. 2010;363(8):711-723. doi:10.1056/NEJMoa1003466 20525992 PMC3549297

[9] Robert C, Thomas L, Bondarenko I, . Ipilimumab plus dacarbazine for previously untreated metastatic melanoma. N Engl J Med. 2011;364(26):2517-2526. doi:10.1056/NEJMoa1104621 21639810

[10] Eggermont AMM, Blank CU, Mandala M, . Adjuvant pembrolizumab versus placebo in resected stage III melanoma. N Engl J Med. 2018;378(19):1789-1801. doi:10.1056/NEJMoa1802357 29658430

[11] Eggermont AM, Chiarion-Sileni V, Grob JJ, . Prolonged survival in stage III melanoma with ipilimumab adjuvant therapy. N Engl J Med. 2016;375(19):1845-1855. doi:10.1056/NEJMoa1611299 27717298 PMC5648545

[12] Weber J, Mandala M, Del Vecchio M, ; CheckMate 238 Collaborators. Adjuvant nivolumab versus ipilimumab in resected stage III or IV melanoma. N Engl J Med. 2017;377(19):1824-1835. doi:10.1056/NEJMoa1709030 28891423

[13] Long GV, Hauschild A, Santinami M, . Adjuvant dabrafenib plus trametinib in stage III BRAF-mutated melanoma. N Engl J Med. 2017;377(19):1813-1823. doi:10.1056/NEJMoa1708539 28891408

[14] Verma V, Sprave T, Haque W, . A systematic review of the cost and cost-effectiveness studies of immune checkpoint inhibitors. J Immunother Cancer. 2018;6(1):128. doi:10.1186/s40425-018-0442-7 30470252 PMC6251215

[15] Vouk K, Benter U, Amonkar MM, . Cost and economic burden of adverse events associated with metastatic melanoma treatments in five countries. J Med Econ. 2016;19(9):900-912. doi:10.1080/13696998.2016.1184155 27123564

[16] Schachter J, Ribas A, Long GV, . Pembrolizumab versus ipilimumab for advanced melanoma: final overall survival results of a multicentre, randomised, open-label phase 3 study (KEYNOTE-006). Lancet. 2017;390(10105):1853-1862. doi:10.1016/S0140-6736(17)31601-X 28822576

[17] Seth R, Messersmith H, Kaur V, . Systemic therapy for melanoma: ASCO guideline. J Clin Oncol. 2020;38(33):3947-3970. doi:10.1200/JCO.20.00198 32228358

[18] Faries MB, Thompson JF, Cochran AJ, . Completion dissection or observation for sentinel-node metastasis in melanoma. N Engl J Med. 2017;376(23):2211-2222. doi:10.1056/NEJMoa1613210 28591523 PMC5548388

[19] Leiter U, Stadler R, Mauch C, ; German Dermatologic Cooperative Oncology Group (DeCOG). Complete lymph node dissection versus no dissection in patients with sentinel lymph node biopsy positive melanoma (DeCOG-SLT): a multicentre, randomised, phase 3 trial. Lancet Oncol. 2016;17(6):757-767. doi:10.1016/S1470-2045(16)00141-8 27161539

[20] Leiter UM, Stadler R, Mauch C, . Final Analysis of DECOG-SLT Trial: Survival Outcomes of Complete Lymph Node Dissection in Melanoma Patients With Positive Sentinel Node. American Society of Clinical Oncology; 2018. 10.1200/JCO.18.02306 31557067

[21] Easson A, Cosby R, McCready D, Temple C, Petrella T, Wright F. *Surgical Management of Patients With Lymph Node Metastases From Cutaneous Melanoma of the Trunk or Extremities*. Ontario Health (Cancer Care Ontario); 2018:8-6.

[22] Gupta A, Eisenhauer EA, Booth CM. The time toxicity of cancer treatment. J Clin Oncol. 2022;40(15):1611-1615. doi:10.1200/JCO.21.02810 35235366

[23] Sedhom R, Samaan A, Gupta A. Caregiver burden #419. J Palliat Med. 2021;24(8):1246-1247. doi:10.1089/jpm.2021.0244 34339334

[24] Porter ME. What is value in health care? N Engl J Med. 2010;363(26):2477-2481. doi:10.1056/NEJMp1011024 21142528

[25] Statistique Canada. Annual Demographic Estimates*: **Provinces and Territories.* Statistique Canada; 2020.

[26] Elder DE, Eguchi MM, Barnhill RL, . Diagnostic error, uncertainty, and overdiagnosis in melanoma. Pathology. 2023;55(2):206-213. doi:10.1016/j.pathol.2022.12.345 36642569 PMC10373372

[27] Mavor ME, Richardson H, Miao Q, Asai Y, Hanna TP. Disparities in diagnosis of advanced melanoma: a population-based cohort study. CMAJ Open. 2018;6(4):E502-E512. doi:10.9778/cmajo.20180089 30381323 PMC6208113

[28] Kralj B; Ontario Medical Association Economics Department. Measuring Rurality—RIO 200_Basic: Methodology and Results. Ontario Medical Association Economics Dept; 2009.

[29] Moore BJ, White S, Washington R, Coenen N, Elixhauser A. Identifying increased risk of readmission and in-hospital mortality using hospital administrative data. Med Care. 2017;55(7):698-705. doi:10.1097/MLR.0000000000000735 28498196

[30] Wodchis WP, Bushmeneva K, Nikitovic M, McKillop I. Guidelines on person-level costing using administrative databases in Ontario. 2013. Accessed July 20, 2023. https://www.scienceopen.com/document?vid=19dd8115-435f-4586-81f3-eaec85280736

[31] Mittmann N, Cheng SY, Liu N, . The generation of two specific cancer costing algorithms using Ontario administrative databases. Curr Oncol. 2019;26(5):e682-e692. doi:10.3747/co.26.5279 31708661 PMC6821127

[32] Mittmann N, Liu N, Cheng SY, . Health system costs for cancer medications and radiation treatment in Ontario for the 4 most common cancers: a retrospective cohort study. CMAJ Open. 2020;8(1):E191-E198. doi:10.9778/cmajo.20190114 32184283 PMC7082106

[33] Austin PC. Optimal caliper widths for propensity-score matching when estimating differences in means and differences in proportions in observational studies. Pharm Stat. 2011;10(2):150-161. doi:10.1002/pst.433 20925139 PMC3120982

[34] Chang CL, Schabert VF, Munakata J, . Comparative healthcare costs in patients with metastatic melanoma in the USA. Melanoma Res. 2015;25(4):312-320. doi:10.1097/CMR.0000000000000159 25882026

[35] Seiger K, Schmults CD, Silk AW, Ruiz ES. Cost and utilization of immunotherapy and targeted therapy for melanoma: cross-sectional analysis in the Medicare population, 2013 and 2015. J Am Acad Dermatol. 2020;82(3):761-764. doi:10.1016/j.jaad.2019.10.023 31626884

[36] Leeneman B, Uyl-de Groot CA, Aarts MJB, . Healthcare costs of metastatic cutaneous melanoma in the era of immunotherapeutic and targeted drugs. Cancers (Basel). 2020;12(4):1003. doi:10.3390/cancers12041003 32325748 PMC7225943

[37] McArthur GA, Mohr P, Ascierto PA, . Health care resource utilization and associated costs among metastatic cutaneous melanoma patients treated with ipilimumab (INTUITION study). Oncologist. 2017;22(8):951-962. doi:10.1634/theoncologist.2016-0272 28526721 PMC5553953

[38] Copley-Merriman C, Stevinson K, Liu FX, . Direct costs associated with adverse events of systemic therapies for advanced melanoma: systematic literature review. Medicine (Baltimore). 2018;97(31):e11736. doi:10.1097/MD.0000000000011736 30075584 PMC6081130

[39] Ghate SR, Li Z, Tang J, Nakasato AR. Economic burden of adverse events associated with immunotherapy and targeted therapy for metastatic melanoma in the elderly. Am Health Drug Benefits. 2018;11(7):334-343. 30647822 PMC6306100

[40] Wehler E, Zhao Z, Pinar Bilir S, Munakata J, Barber B. Economic burden of toxicities associated with treating metastatic melanoma in eight countries. Eur J Health Econ. 2017;18(1):49-58. doi:10.1007/s10198-015-0757-y 26721505 PMC5209401

[41] Almutairi AR, Alkhatib NS, Oh M, . Economic evaluation of talimogene laherparepvec plus ipilimumab combination therapy vs ipilimumab monotherapy in patients with advanced unresectable melanoma. JAMA Dermatol. 2019;155(1):22-28. doi:10.1001/jamadermatol.2018.3958 30477000 PMC6439581

[42] Lu B, Dai WF, Croxford R, . Cost-effectiveness of second-line ipilimumab for metastatic melanoma: a real-world population-based cohort study of resource utilization. Cancer Med. 2023;12(10):11451-11461. doi:10.1002/cam4.5862 36999965 PMC10242360

[43] Paly VF, Hikichi Y, Baker T, Itakura E, Chandran N, Harrison J. Economic evaluation of nivolumab combined with ipilimumab in the first-line treatment of advanced melanoma in Japan. J Med Econ. 2020;23(12):1542-1552. doi:10.1080/13696998.2020.1830781 33000994

[44] Rubio-Rodríguez D, De Diego Blanco S, Pérez M, Rubio-Terrés C. Cost-effectiveness of drug treatments for advanced melanoma: a systematic literature review. Pharmacoeconomics. 2017;35(9):879-893. doi:10.1007/s40273-017-0517-1 28551858

[45] Gorry C, McCullagh L, Barry M. Economic evaluation of systemic treatments for advanced melanoma: a systematic review. Value Health. 2020;23(1):52-60. doi:10.1016/j.jval.2019.07.003 31952674

[46] Conic RZ, Cabrera CI, Khorana AA, Gastman BR. Determination of the impact of melanoma surgical timing on survival using the National Cancer Database. J Am Acad Dermatol. 2018;78(1):40-46.e7. doi:10.1016/j.jaad.2017.08.039 29054718 PMC6053055

[47] Neal RD, Tharmanathan P, France B, . Is increased time to diagnosis and treatment in symptomatic cancer associated with poorer outcomes? Systematic review. Br J Cancer. 2015;112(suppl 1):S92-S107. doi:10.1038/bjc.2015.48 25734382 PMC4385982

[48] Hanna TP, Nguyen P, Baetz T, Booth CM, Eisenhauer E. A population-based study of survival impact of new targeted and immune-based therapies for metastatic or unresectable melanoma. Clin Oncol (R Coll Radiol). 2018;30(10):609-617. doi:10.1016/j.clon.2018.05.005 30196844

[49] Adams S, Gatti-Mays ME, Kalinsky K, . Current landscape of immunotherapy in breast cancer: a review. JAMA Oncol. 2019;5(8):1205-1214. doi:10.1001/jamaoncol.2018.7147 30973611 PMC8452050

[50] Reck M, Remon J, Hellmann MD. First-line immunotherapy for non–small-cell lung cancer. J Clin Oncol. 2022;40(6):586-597. doi:10.1200/JCO.21.01497 34985920

[51] Sharma P, Siefker-Radtke A, de Braud F, . Nivolumab alone and with ipilimumab in previously treated metastatic urothelial carcinoma: CheckMate 032 nivolumab 1 mg/kg plus ipilimumab 3 mg/kg expansion cohort results. J Clin Oncol. 2019;37(19):1608-1616. doi:10.1200/JCO.19.00538 31100038 PMC6879315

[52] André T, Shiu KK, Kim TW, ; KEYNOTE-177 Investigators. Pembrolizumab in microsatellite-instability–high advanced colorectal cancer. N Engl J Med. 2020;383(23):2207-2218. doi:10.1056/NEJMoa2017699 33264544

