# Supplementary material for: Changes in Health Care Costs, Survival, and Time Toxicity in the Era of Immunotherapy and Targeted Systemic Therapy for Melanoma
Source: JAMA Dermatol. 2023 Sep 6;159(11):1195–204. doi: 10.1001/jamadermatol.2023.3179 (PMC10483386; doi:10.1001/jamadermatol.2023.3179)
Supplement: Supplement 1. — eFigure 1. Flow Diagram for Invasive Cutaneous Melanoma Patients and Their Pathology Records in Ontario From January 1, 2007 to December 31, 2012 eFigure 2. Flow Diagram for Invasive Cutaneous Melanoma Patients in Ontario From January 1, 2018 to March 31, 2019 eFigure 3. Flow Diagram for Propensity Score Matched Stage II-IV Melanoma Patients in Ontario From January 1, 2007 to December 31, 2012 and January 1, 2018 to March 31, 2019 eFigure 4. Kaplan-Meier Survival Curves for the Unmatched Cohorts Stratified by Stage eAppendix. Description of ICES Administrative Data Sources eTable 1. Patient Characteristics for Unmatched and Matched Cohorts of Stage II Patients eTable 2. Patient Characteristics for Unmatched and Matched Cohorts of Stage III Patients eTable 3. Patient Characteristics for Unmatched and Matched Cohorts of Stage IV Patients eTable 4. Mean Healthcare Utilization Per-Person Costs in Canadian Dollars for the Unmatched Cohorts Stratified by Stage eTable 5. Mean Systemic Therapy Per-Person Costs in Canadian Dollars for the Unmatched Cohorts Stratified by Stage eTable 6. Mean Systemic Therapy Per-Person Costs in Canadian Dollars for the Matched Cohorts Stratified by Stage eTable 7. Time Toxicity Sensitivity Analysis in Days Including Virtual Visits and Home Care Visits [file jamadermatol-e233179-s001.pdf]

Supplementary Online Content

Bateni SB, Nguyen P, Eskander A, et al. Changes in health care costs, survival, and time toxicity in the era of immunotherapy and targeted systemic therapy for melanoma. *JAMA Dermatol.* Published online September 6, 2023. doi:10.1001/jamadermatol.2023.3179

eFigure 1. Flow Diagram for Invasive Cutaneous Melanoma Patients and Their Pathology Records in Ontario From January 1, 2007 to December 31, 2012

eFigure 2. Flow Diagram for Invasive Cutaneous Melanoma Patients in Ontario From January 1, 2018 to March 31, 2019

eFigure 3. Flow Diagram for Propensity Score Matched Stage II-IV Melanoma Patients in Ontario From January 1, 2007 to December 31, 2012 and January 1, 2018 to March 31, 2019

eFigure 4. Kaplan-Meier Survival Curves for the Unmatched Cohorts Stratified by Stage

eAppendix. Description of ICES Administrative Data Sources

eTable 1. Patient Characteristics for Unmatched and Matched Cohorts of Stage II Patients

eTable 2. Patient Characteristics for Unmatched and Matched Cohorts of Stage III Patients

eTable 3. Patient Characteristics for Unmatched and Matched Cohorts of Stage IV Patients

eTable 4. Mean Healthcare Utilization Per-Person Costs in Canadian Dollars for the Unmatched Cohorts Stratified by Stage

eTable 5. Mean Systemic Therapy Per-Person Costs in Canadian Dollars for the Unmatched Cohorts Stratified by Stage

eTable 6. Mean Systemic Therapy Per-Person Costs in Canadian Dollars for the Matched Cohorts Stratified by Stage

eTable 7. Time Toxicity Sensitivity Analysis in Days Including Virtual Visits and Home Care Visits

This supplementary material has been provided by the authors to give readers additional information about their work.

© 2023 Bateni SB et al. *JAMA Dermatology.*

eFigure 1. Flow diagram for invasive cutaneous melanoma patients and their pathology records in Ontario from January 1, 2007 to December 31, 2012.

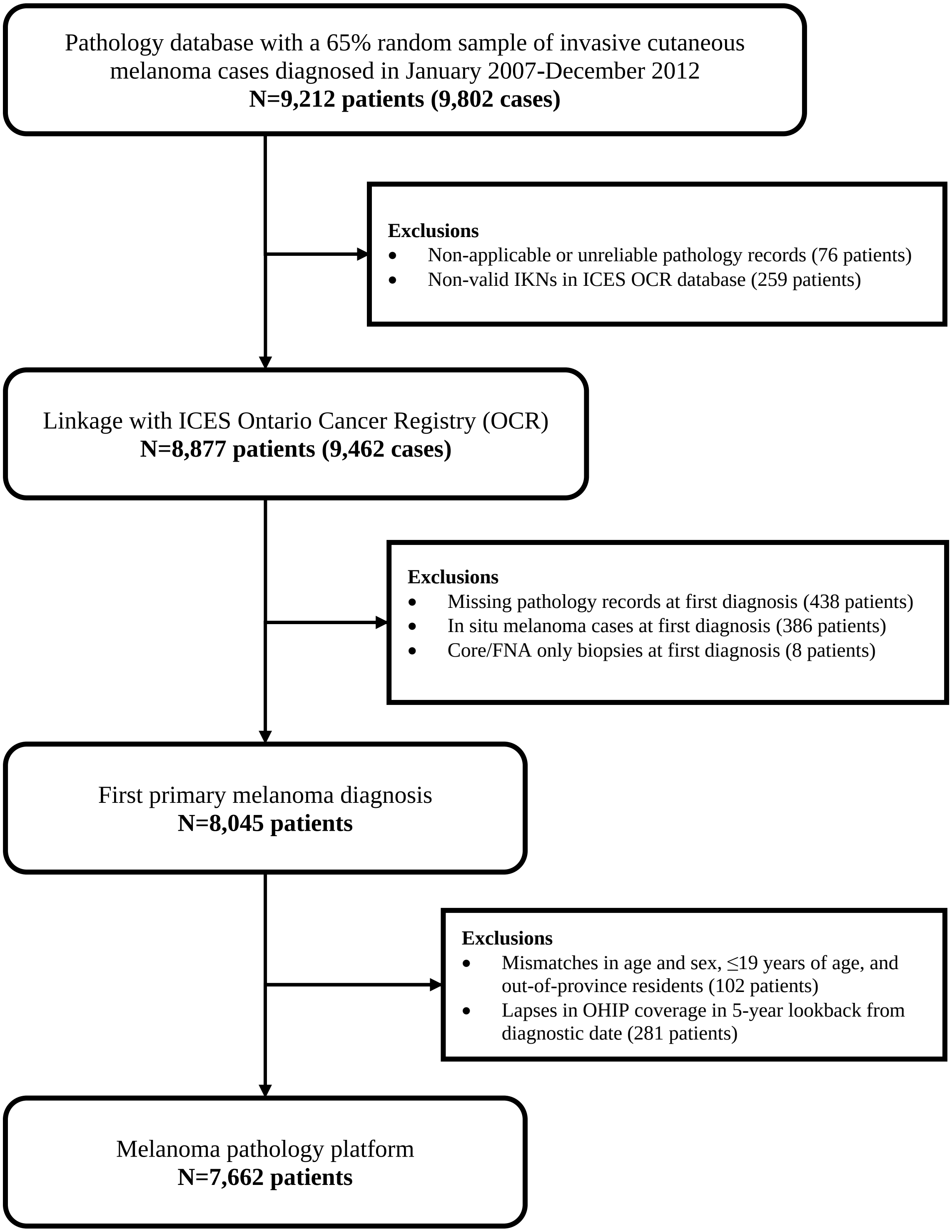

© 2023 Bateni SB et al. *JAMA Dermatology.*

eFigure 2. Flow diagram for invasive cutaneous melanoma patients in Ontario from January 1, 2018 to March 31, 2019.

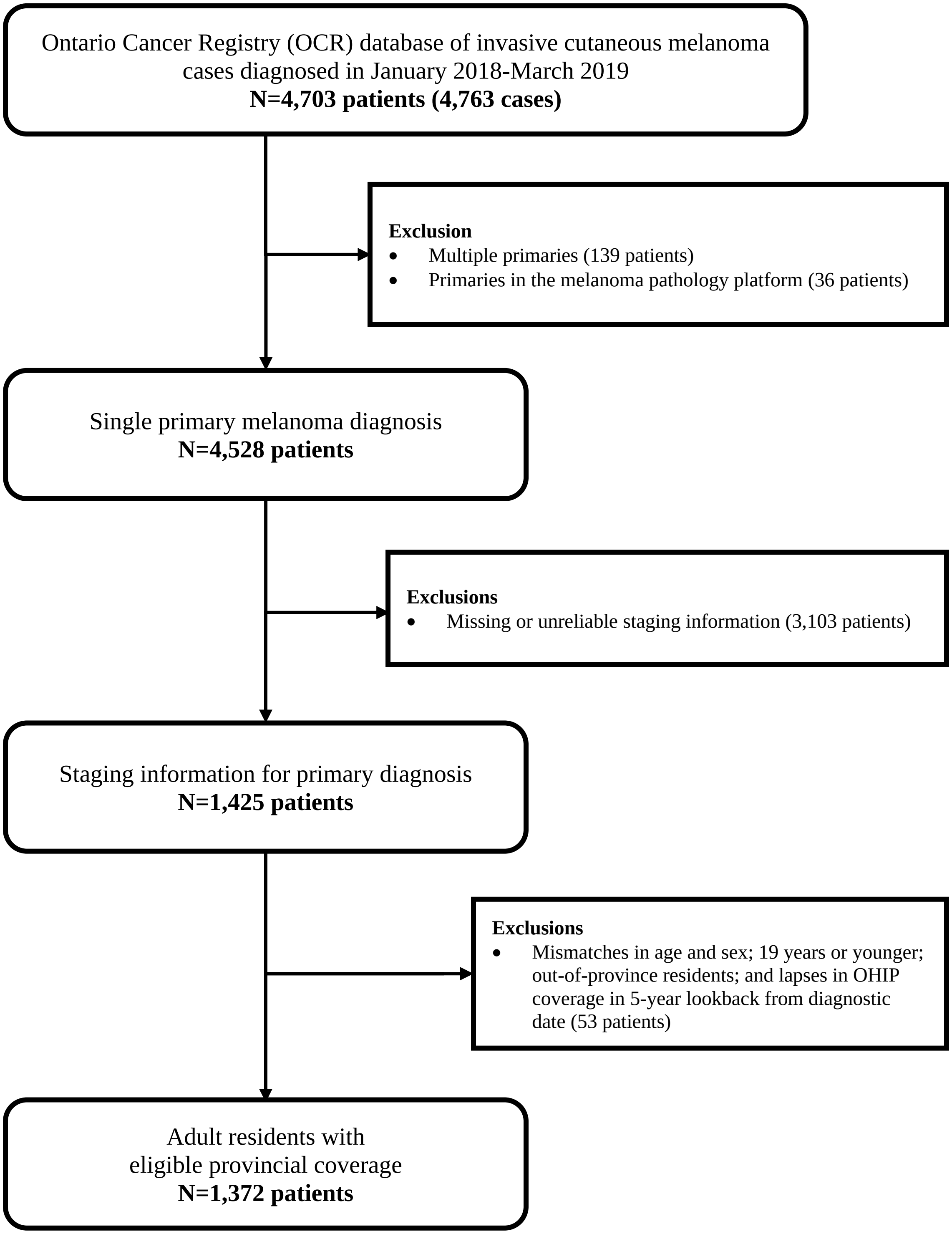

© 2023 Bateni SB et al. *JAMA Dermatology.*

eFigure 3. Flow diagram for propensity score matched Stage II-IV melanoma patients in Ontario from January 1, 2007 to December 31, 2012 and January 1, 2018 to March 31, 2019.

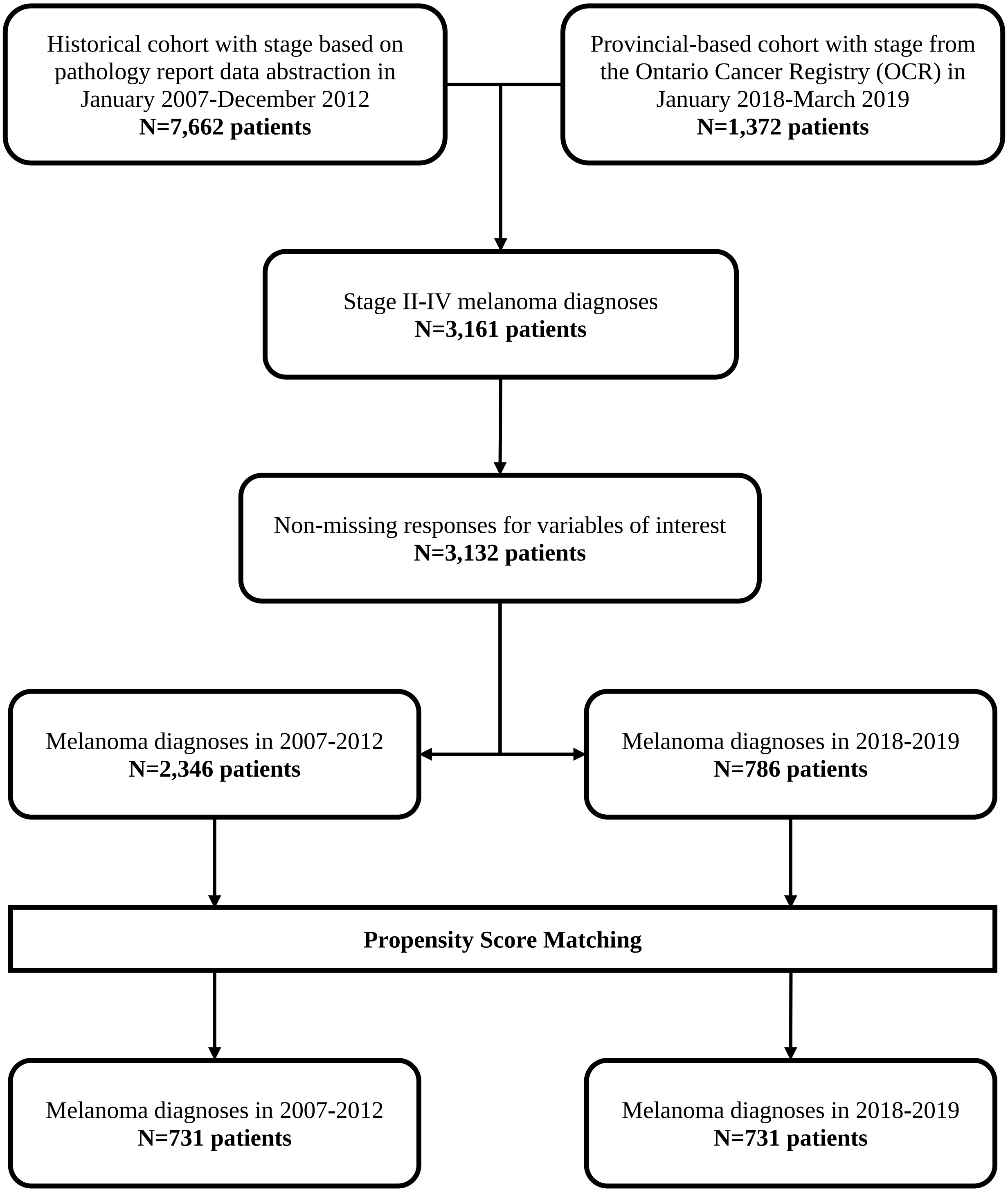

© 2023 Bateni SB et al. *JAMA Dermatology.*

eFigure 4: Kaplan-Meier survival curves for the unmatched cohorts stratified by stage.

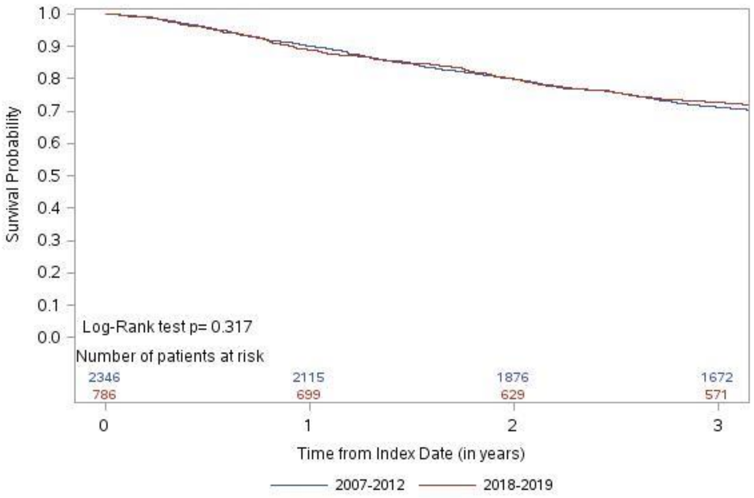

(A) All patients

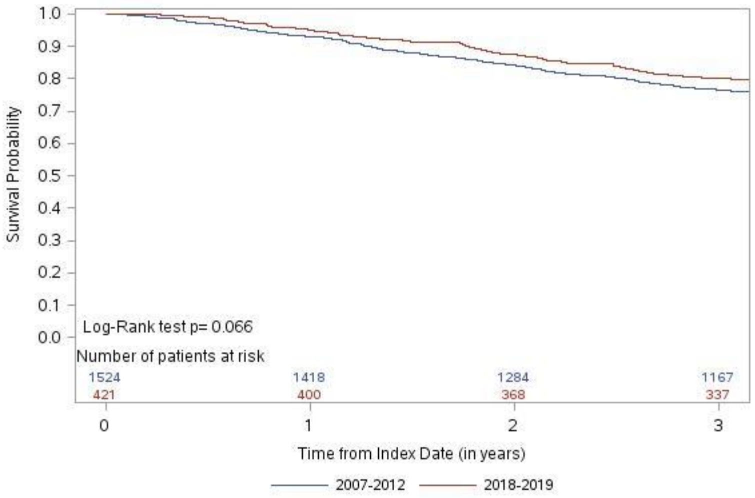

(B) Stage II patients

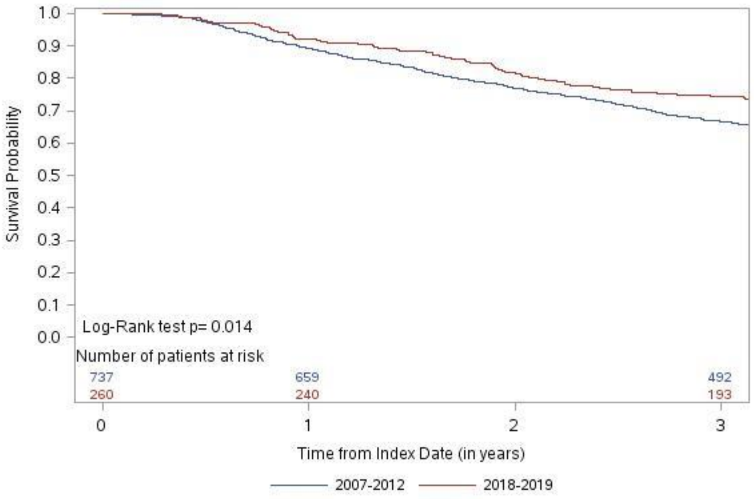

(C) Stage III patients*

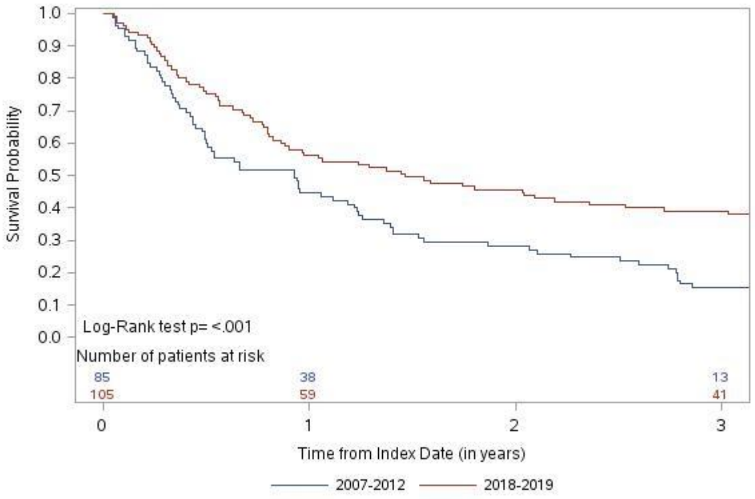

(D) Stage IV patients*

*At Year 2, the number of patients at risk was not shown due to privacy regulations for groups of ≤5 patients.

© 2023 Bateni SB et al. *JAMA Dermatology.*

eAppendix. Description of ICES administrative data sources.

Ontario Health Insurance Plan (OHIP) Claims History Database

The OHIP claims database contains information on inpatient and outpatient services provided to Ontario residents eligible for the province's publicly funded health insurance system by fee-for-service health care practitioners (primarily physicians) and "shadow billings" for those paid through non-fee-for-service payment plans.

Billing codes on the claims (OHIP fee codes) identify the care provider, their area of specialization and the type and location of service. OHIP billing claims also contain a 3-digit diagnosis code - the main reason for the service - captured using a modified version of the ICD, 8th revision coding system. OHIP claims are well completed, but the validity of the diagnosis coding is highly variable.

Discharge Abstract Database (DAD)

The DAD is compiled by the Canadian Institute for Health Information (CIHI) and contains administrative, clinical (diagnoses and procedures/interventions), demographic, and administrative information for all admissions to acute care hospitals in Ontario. At ICES, consecutive DAD records are linked together to form "episodes of care" among the hospitals to which patients have been transferred after their initial admission.

Prior to April 1, 2002, diagnoses (up to 16 on a given DAD record) are captured using the International Statistical Classification of Diseases, Injuries, and Causes of Death, 9th Revision (ICD-9) coding system and procedures (up to 10 on a given DAD record) are captured using the Canadian Classification of Diagnostic, Therapeutic, and Surgical Procedures (CCP) coding system. Following April 1, 2002, diagnoses (up to 25 on a given DAD record) are captured using the International Statistical Classification of Diseases and Related Health Problems, 10th Revision, Canada (ICD-10-CA) coding system and interventions (up to 20 on a given DAD record) are captured using the Canadian Classification of Health Interventions (CCI) coding system.

Ontario Mental Health Reporting System (OMHRS)

The OMHRS is compiled by CIHI and contains administrative, clinical (diagnoses and procedures), demographic, and administrative information for all admissions to adult designated inpatient mental health beds. This includes beds in general hospitals, provincial psychiatric facilities, and specialty psychiatric facilities. Clinical assessment data is ascertained using the Resident Assessment Instrument for Mental Health (RAI-MH), but different amounts of information are collected using this instrument depending on the length of stay in the mental health bed. Multiple assessments may occur during the length of a mental health admission.

Prior to April 1, 2016, psychiatric diagnoses are captured using the Diagnostic and Statistical Manual of Mental Disorders, 4th Edition, Text Revision (DSM-IV-TR) coding system. Following April 1, 2016, psychiatric diagnoses are captured using the Diagnostic and Statistical Manual of Mental Disorders, 5th Edition, Text Revision (DSM-V-TR) coding system. Non-psychiatric diagnoses are captured using the ICD-10-CA coding system.

© 2023 Bateni SB et al. *JAMA Dermatology.*

Same Day Surgery (SDS) Database

The SDS is compiled by CIHI and contains administrative, clinical (diagnoses and procedures), demographic, and administrative information for all patient visits made to day surgery institutions in Ontario.

Prior to April 1, 2002, diagnoses (up to 16 on a given SDS record) were captured using the ICD-9 coding system and procedures (up to 10 on a given SDS record) were captured using the CCP coding system. Since April 1, 2002, diagnoses (up to 25 on a given SDS record) are captured using the ICD-10-CA coding system and interventions (up to 16 on a given SDS record) are captured using the CCI coding system.

National Ambulatory Care Reporting System (NACRS)

The NACRS is compiled by CIHI and contains administrative, clinical (diagnoses and procedures), demographic, and administrative information for all patient visits made to hospital- and community-based ambulatory care centers (emergency departments, day surgery units, hemodialysis units, and cancer care clinics) in Ontario. At ICES, NACRS records are linked with other data sources (DAD, OMHRS) to identify transitions to other care settings, such as inpatient acute care or psychiatric care.

Prior to April 1, 2002, diagnoses (up to 6 on a given NACRS record) are captured using the ICD-9 coding system and procedures (up to 10 on a given NACRS record) are captured using the CCP coding system. Following April 1, 2002, diagnoses (up to 10 on a given NACRS record) are captured using the ICD-10-CA coding system and interventions (up to 10 on a given NACRS record) are captured using the CCI coding system. NACRS emergency department diagnosis codes have been extensively validated.

Continuing Care Reporting System (CCRS)

The CCRS database is compiled by CIHI and contains demographic, clinical, functional, and resource utilization information for individuals receiving facility-based continuing care (also known as extended, auxiliary, or complex chronic care) in Ontario hospitals. Clinical assessment data (on the physical, functional, cognitive, and social domains of health) is ascertained using the Resident Assessment Instrument Minimum Data Set (RAI-MDS) version 2.0 which is administered by trained healthcare professionals.

National Rehabilitation Reporting System (NRS)

The NRS contains patient data collected from participating adult inpatient rehabilitation facilities and programs across Canada. NRS collects data for the purposes of supporting CIHI's mandate, collecting processing and analyzing adult inpatient rehabilitation services, supporting management decision making at the hospital, facilitating provincial and national comparative reporting and supporting related approved analysis and research.

Home Care Database (HCD)

The HCD is maintained by the Health System Performance and Support Division of Ontario Health, formerly Health Shared Services Ontario. HCD is a clinical, client-centered database that captures all home care services provided or coordinated by Local Health Integration Networks. This dataset contains information on client, intake, assessment, admission, diagnostic and surgical procedure, and service delivery. Data are collected for the purpose of helping home care clinicians code diseases and other health conditions that frequently occur in home care.

Ontario Cancer Registry (OCR)

© 2023 Bateni SB et al. *JAMA Dermatology.*

The OCR is a computerized database of information on all Ontario residents who have been newly diagnosed with cancer since 1964. All new cases of cancer, except non-melanoma skin cancer, are registered in the information system which is managed and maintained by Ontario Health (Cancer Care Ontario). Data from multiple sources, including DAD and SDS records from CIHI which include a diagnosis of cancer, paper reports from pathology departments with any mention of cancer, electronic reports from the Ontario regional cancer centers and Princess Margaret Hospital (the specialized institutions treated cancer patients in Ontario), and electronic reports of all deaths of Ontario residents from the Office of the Registrar General of Ontario based on Ontario Provincial death certificates with cancer as the underlying cause of death are linked to compile incident cases of cancer in Ontario. Approximately 95% of all diagnosed cancer cases in Ontario are captured by the OCR.

Symptom Management Database (ESAS)

The ESAS database is a web-based symptom screening tool provided by Ontario Health (Cancer Care Ontario) to healthcare providers (and their patients where available) to monitor patients symptoms. Data are collected for the purpose of improving symptom management and collaborative palliative care planning through earlier identification, documentation and communication of patient symptoms and performance status. This data holding consists of four smaller datasets: Edmonton Symptom Assessment System (ESAS), Eastern Cooperative Oncology Group Performance Status (ECOG), Patient Reported Functional Status (PRFS) and Palliative Performance Scale (PPS).

Cancer Activity Level Reporting (ALR)

The ALR dataset represents the basic set of data elements required to produce the quality, cost and performance indicators for the provincial cancer system. The data elements constitute patient level activity within the cancer system focused on radiation and systemic therapy services and outpatient oncology clinic visits. This data is also a key component of the OCR, which registers every malignant neoplasm diagnosed in Ontario.

A Regional Cancer Program encompasses the delivery of all cancer services regardless of where that service is delivered in the facility. Ontario Health (Cancer Care Ontario) requires all activity from the cancer center and its related outreach clinics, if any. In addition, all outpatient chemotherapy treatment visits that occur in the host hospitals are included.

New Drug Funding Program (NDFP)

The NDFP is one of four publicly funded drug programs under the Ontario Public Drug Programs. Administered by Ontario Health (Cancer Care Ontario), the data collected relates to the funding of new and often expensive cancer drugs for the purpose of ensuring that Ontario patients have equal access to high-quality intravenous (IV) cancer drugs provided at hospitals and cancer centres.

Ontario Drug Benefit (ODB) Program

The ODB database contains prescription medication claims for those covered under the provincial drug program, mainly: those aged 65 years and older, nursing home residents, patients receiving services under the Ontario Home Care program, those receiving social assistance, and residents eligible for specialized drug programs (e.g., programs which cover the costs of medications for individuals whose medication costs exceed 4% of their net household income and for individuals with rare, serious conditions).

© 2023 Bateni SB et al. *JAMA Dermatology.*

Each medication claim has an associated prescriber identifier which indicates the health practitioner who wrote the prescription, as well as fields that identify the type and quantity of medication and duration of treatment. A special flag in the ODB database indicates whether the prescription was dispensed to a nursing home resident.

Registered Persons Database (RPDB)

The RPDB provides basic demographic information (age, sex, location of residence, date of birth, and date of death for deceased individuals) for those issued an Ontario health insurance number. The RPDB also indicates the time periods for which an individual was eligible to receive publicly funded health insurance benefits and the best known postal code for each registrant on July 1st of each year.

ICES Physician Database (IPDB)

The IPDB provides information about all physicians who have practiced in Ontario and is comprised of data contained in the OHIP claims history database, the OHIP Corporate Provider Database (CPDB), and the Ontario Physician Human Resource Data Centre (OPHRDC) database. The database contains information on demographics (age, gender, year of graduation, school of graduation); specialty (functional and certified); location of practice; and measures of physician activity (billings and workload data).

Client Agency Program Enrollment (CAPE)

The CAPE database is a registry of all patients who have ever been rostered to receive care from a particular physician in Ontario and documents the time period in which a patient was rostered to a specific physician.

Descriptions were drawn from the ICES Intranet with adaption from the following references:

1. ICES. ICES data dictionary. https://datadictionary.ices.on.ca. Accessed January 10, 2023. 2023.
2. ICES Privacy & Legal Office. ICES Report to the Information and Privacy Commissioner of Ontario: Three-Year Review as a Prescribed Entity under PHIPA, 2020.

© 2023 Bateni SB et al. *JAMA Dermatology.*

eTable 1. Patient characteristics for unmatched and matched cohorts of Stage II patients.

Patient Characteristics	Unmatched		Std. Diff.[a]	Matched		Std. Diff.[a]
	2007-2012 (N=1,524)	2018-2019 (N=421)		2007-2012 (N=421)	2018-2019 (N=421)	
**Age (mean ± SD)**	68.7 ± 15.3	70.2 ± 14.5	0.10	70.0 ± 13.9	70.2 ± 14.5	0.01
**Age (categorized)**						
20-39	69 (4.5%)	13 (3.1%)	0.08	13 (3.1%)	13 (3.1%)	0.00
40-49	116 (7.6%)	18 (4.3%)	0.14	18 (4.3%)	18 (4.3%)	0.00
50-59	212 (13.9%)	69 (16.4%)	0.07	66 (15.7%)	69 (16.4%)	0.02
60-69	317 (20.8%)	96 (22.8%)	0.05	93 (22.1%)	96 (22.8%)	0.02
70-79	389 (25.5%)	94 (22.3%)	0.07	109 (25.9%)	94 (22.3%)	0.08
80+	421 (27.6%)	131 (31.1%)	0.08	122 (29.0%)	131 (31.1%)	0.05
**Sex**						
Female	618 (40.6%)	181 (43.0%)	0.05	172 (40.9%)	181 (43.0%)	0.04
Male	906 (59.4%)	240 (57.0%)	0.05	249 (59.1%)	240 (57.0%)	0.04
**Income quintile**						
1 (Lowest)	248 (16.3%)	71 (16.9%)	0.02	70 (16.6%)	71 (16.9%)	0.01
2	276 (18.1%)	88 (20.9%)	0.07	85 (20.2%)	88 (20.9%)	0.02
3	311 (20.4%)	91 (21.6%)	0.03	88 (20.9%)	91 (21.6%)	0.02
4	333 (21.9%)	74 (17.6%)	0.11	78 (18.5%)	74 (17.6%)	0.02
5 (Highest)	356 (23.4%)	97 (23.0%)	0.01	100 (23.8%)	97 (23.0%)	0.02
**Place of residence**						
Erie St. Clair	99 (6.5%)	25 (5.9%)	0.02	28 (6.7%)	25 (5.9%)	0.03
South West	151 (9.9%)	36 (8.6%)	0.05	43 (10.2%)	36 (8.6%)	0.06
Waterloo Wellington	92 (6.0%)	30 (7.1%)	0.04	35 (8.3%)	30 (7.1%)	0.04
HNHB	237 (15.6%)	52 (12.4%)	0.09	58 (13.8%)	52 (12.4%)	0.04
Central West	50 (3.3%)	13 (3.1%)	0.01	15 (3.6%)	13 (3.1%)	0.03
Mississauga Halton	96 (6.3%)	18 (4.3%)	0.09	20 (4.8%)	18 (4.3%)	0.02
Toronto Central	107 (7.0%)	42 (10.0%)	0.11	28 (6.7%)	42 (10.0%)	0.12
Central	137 (9.0%)	50 (11.9%)	0.09	43 (10.2%)	50 (11.9%)	0.05
Central East	194 (12.7%)	56 (13.3%)	0.02	51 (12.1%)	56 (13.3%)	0.04
South East	94 (6.2%)	24 (5.7%)	0.02	29 (6.9%)	24 (5.7%)	0.05
Champlain	101 (6.6%)	34 (8.1%)	0.06	30 (7.1%)	34 (8.1%)	0.04
North Simcoe Muskoka	64 (4.2%)	23 (5.5%)	0.06	19 (4.5%)	23 (5.5%)	0.04
North East/North West	102 (6.7%)	18 (4.3%)	0.11	22 (5.2%)	18 (4.3%)	0.04
**Urban/rural residence**						
Urban	934 (61.3%)	268 (63.7%)	0.05	251 (59.6%)	268 (63.7%)	0.08
Suburban	429 (28.2%)	111 (26.4%)	0.04	125 (29.7%)	111 (26.4%)	0.07
Rural	161 (10.6%)	42 (10.0%)	0.02	45 (10.7%)	42 (10.0%)	0.02
**Elixhauser comorbidity index (mean ± SD)**	0.66 ± 1.32	0.65 ± 1.32	0.01	0.69 ± 1.34	0.65 ± 1.32	0.03
**Elixhauser comorbidity index (categorized)**						
0-1	1,272 (83.5%)	349 (82.9%)	0.02	349 (82.9%)	349 (82.9%)	0.00
2-3	172 (11.3%)	52 (12.4%)	0.03	50 (11.9%)	52 (12.4%)	0.01
4+	80 (5.3%)	20 (4.8%)	0.02	22 (5.2%)	20 (4.8%)	0.02
**Histology (categorized)**						
Melanoma, NOS	347 (22.8%)	133 (31.6%)	0.20	147 (34.9%)	133 (31.6%)	0.07

© 2023 Bateni SB et al. *JAMA Dermatology.*

Nodular melanoma	564 (37.0%)	158 (37.5%)	0.01	147 (34.9%)	158 (37.5%)	0.05
Lentigo maligna/acral lentiginous melanoma	69 (4.5%)	28 (6.7%)	0.09	21 (5.0%)	28 (6.7%)	0.07
Superficial spreading melanoma	331 (21.7%)	66 (15.7%)	0.16	72 (17.1%)	66 (15.7%)	0.04
Other	213 (14.0%)	36 (8.6%)	0.17	34 (8.1%)	36 (8.6%)	0.02
**Body site (categorized)**						
Head/neck	367 (24.1%)	93 (22.1%)	0.05	92 (21.9%)	93 (22.1%)	0.01
Trunk	453 (29.7%)	120 (28.5%)	0.03	125 (29.7%)	120 (28.5%)	0.03
Arm/shoulder	386 (25.3%)	136 (32.3%)	0.15	114 (27.1%)	136 (32.3%)	0.11
Leg/hip/other	318 (20.9%)	72 (17.1%)	0.10	90 (21.4%)	72 (17.1%)	0.11
**Treatment**						
Primary surgery[b]						
WLE	875 (57.4%)	210 (49.9%)	0.15	249 (59.1%)	210 (49.9%)	0.19
Amputation	40 (2.6%)	8 (1.9%)	0.05	17 (4.0%)	8 (1.9%)	0.13
Graft	116 (7.6%)	43 (10.2%)	0.09	34 (8.1%)	43 (10.2%)	0.07
Flap	316 (20.7%)	126 (29.9%)	0.21	74 (17.6%)	126 (29.9%)	0.29
Other excisions	110 (7.2%)	22 (5.2%)	0.08	25 (5.9%)	22 (5.2%)	0.03
None	67 (4.4%)	12 (2.9%)	0.08	22 (5.2%)	12 (2.9%)	0.12
Nodal surgery						
SLNB	897 (58.9%)	274 (65.1%)	0.13	241 (57.2%)	274 (65.1%)	0.16
CLND	162 (10.6%)	21 (5.0%)	0.21	47 (11.2%)	21 (5.0%)	0.23
Systemic therapy	149 (9.8%)	58 (13.8%)	0.12	43 (10.2%)	58 (13.8%)	0.11
Radiotherapy	70 (4.6%)	26 (6.2%)	0.07	22 (5.2%)	26 (6.2%)	0.04
Metastasis surgery[c]	0 (0.00%)	≤5 (1.2%)	N/A	0 (0.00%)	≤5 (1.2%)	N/A

Std. Diff., standardized differences; SD, standard deviation; HNHB, Hamilton Niagara Haldimand Brant; NOS, not otherwise specified; WLE, wide local excision; SLNB, sentinel lymph node biopsy; CLND, completion lymph node dissection.

[a]A standardized difference of ≥0.20 suggests a significant effect size between groups. [b]If more than one type of primary surgery occurred, treatment is prioritized as follows: Amputation > WLE > graft > flap > other excision. [c]Metastasis surgery includes operations for spinal cord, brain, lung, and liver metastases. [d]Categorical responses with ≤5 patients were suppressed due to privacy regulations.

© 2023 Bateni SB et al. *JAMA Dermatology.*

eTable 2. Patient characteristics for unmatched and matched cohorts of Stage III patients.

Patient Characteristics	Unmatched		Std. Diff.[a]	Matched		Std. Diff.[a]
	2007-2012 (N=737)	2018-2019 (N=260)		2007-2012 (N=254)	2018-2019 (N=254)	
**Age (mean ± SD)**	61.5 ± 16.5	63.6 ± 14.2	0.13	64.3 ± 14.4	63.5 ± 14.4	0.05
**Age (categorized)**						
20-39	81 (11.0%)	≤39 (15.0%)	N/A	≤35 (13.8%)	≤65 (25.6%)	N/A
40-49	103 (14.0%)	≤39 (15.0%)	N/A	≤35 (13.8%)	21 (8.3%)	N/A
50-59	134 (18.2%)	47 (18.1%)	0.00	55 (21.7%)	≤65 (25.6%)	N/A
60-69	160 (21.7%)	81 (31.2%)	0.22	70 (27.6%)	75 (29.5%)	0.04
70-79	141 (19.1%)	65 (25.0%)	0.14	52 (20.5%)	65 (25.6%)	0.12
80+	118 (16.0%)	28 (10.8%)	0.15	42 (16.5%)	28 (11.0%)	0.16
**Sex**						
Female	290 (39.3%)	89 (34.2%)	0.11	107 (42.1%)	87 (34.3%)	0.16
Male	447 (60.7%)	171 (65.8%)	0.11	147 (57.9%)	167 (65.7%)	0.16
**Income quintile**						
1 (Lowest)	139 (18.9%)	44 (16.9%)	0.05	42 (16.5%)	44 (17.3%)	0.02
2	154 (20.9%)	41 (15.8%)	0.13	54 (21.3%)	39 (15.4%)	0.15
3	123 (16.7%)	48 (18.5%)	0.05	45 (17.7%)	46 (18.1%)	0.01
4	149 (20.2%)	60 (23.1%)	0.07	45 (17.7%)	60 (23.6%)	0.15
5 (Highest)	172 (23.3%)	67 (25.8%)	0.06	68 (26.8%)	65 (25.6%)	0.03
**Place of residence**						
Erie St. Clair	42 (5.7%)	19 (7.3%)	0.07	16 (6.3%)	19 (7.5%)	0.05
South West	73 (9.9%)	38 (14.6%)	0.14	25 (9.8%)	36 (14.2%)	0.13
Waterloo Wellington	61 (8.3%)	25 (9.6%)	0.05	27 (10.6%)	25 (9.8%)	0.03
HNHB	116 (15.7%)	26 (10.0%)	0.17	34 (13.4%)	26 (10.2%)	0.10
Central West	23 (3.1%)	7 (2.7%)	0.03	6 (2.4%)	7 (2.8%)	0.02
Mississauga Halton	57 (7.7%)	9 (3.5%)	0.19	11 (4.3%)	9 (3.5%)	0.04
Toronto Central	54 (7.3%)	11 (4.2%)	0.13	21 (8.3%)	11 (4.3%)	0.16
Central	53 (7.2%)	19 (7.3%)	0.00	21 (8.3%)	19 (7.5%)	0.03
Central East	85 (11.5%)	26 (10.0%)	0.05	24 (9.5%)	26 (10.2%)	0.03
South East	47 (6.4%)	18 (6.9%)	0.02	16 (6.3%)	18 (7.1%)	0.03
Champlain	49 (6.7%)	36 (13.9%)	0.24	24 (9.5%)	32 (12.6%)	0.10
North Simcoe Muskoka	44 (6.0%)	10 (3.9%)	0.10	19 (7.5%)	10 (3.9%)	0.15
North East/North West	33 (4.5%)	16 (6.2%)	0.07	10 (3.9%)	16 (6.3%)	0.11
**Urban/rural residence**						
Urban	460 (62.4%)	136 (52.3%)	0.21	153 (60.2%)	132 (52.0%)	0.17
Suburban	202 (27.4%)	92 (35.4%)	0.17	≤101 (39.8%)	92 (36.2%)	N/A
Rural	75 (10.2%)	32 (12.3%)	0.07	≤101 (39.8%)	30 (11.8%)	N/A
**Elixhauser comorbidity index (mean ± SD)**	0.54 ± 1.22	0.47 ± 1.13	0.06	0.55 ± 1.08	0.48 ± 1.14	0.06
**Elixhauser comorbidity index (categorized)**						
0-1	638 (86.6%)	231 (88.9%)	0.07	215 (84.7%)	225 (88.6%)	0.12
2-3	68 (9.2%)	23 (8.9%)	0.01	30 (11.8%)	23 (9.1%)	0.09

© 2023 Bateni SB et al. *JAMA Dermatology.*

4+	31 (4.2%)	6 (2.3%)	0.11	9 (3.5%)	6 (2.4%)	0.07
**Histology (categorized)**						
Melanoma, NOS	168 (22.8%)	108 (41.5%)	0.41	107 (42.1%)	102 (40.2%)	0.04
Nodular melanoma	245 (33.2%)	77 (29.6%)	0.08	75 (29.5%)	77 (30.3%)	0.02
Lentigo maligna/acral lentiginous melanoma	≤141 (19.1%)	≤23 (8.9%)	N/A	≤25 (9.8%)	≤23 (9.1%)	N/A
Superficial spreading melanoma	183 (24.8%)	52 (20.0%)	0.12	47 (18.5%)	52 (20.5%)	0.05
Other	≤141 (19.1%)	≤23 (8.9%)	N/A	≤25 (9.8%)	≤23 (9.1%)	N/A
**Body site (categorized)**						
Head/neck	113 (15.3%)	34 (13.1%)	0.06	36 (14.2%)	34 (13.4%)	0.02
Trunk	262 (35.6%)	97 (37.3%)	0.04	91 (35.8%)	95 (37.4%)	0.03
Arm/shoulder	161 (21.9%)	48 (18.5%)	0.08	54 (21.3%)	45 (17.7%)	0.09
Leg/hip/other	201 (27.3%)	81 (31.2%)	0.09	73 (28.7%)	80 (31.5%)	0.06
**Treatment**						
Primary surgery[b]						
WLE	495 (67.2%)	138 (53.1%)	0.29	175 (68.9%)	134 (52.8%)	0.34
Amputation	≤74 (10.1%)	≤28 (10.8%)	N/A	≤27 (10.6%)	≤28 (11.0%)	N/A
Graft	≤74 (10.1%)	≤28 (10.8%)	N/A	≤27 (10.6%)	≤28 (11.0%)	N/A
Flap	145 (19.7%)	69 (26.5%)	0.16	45 (17.7%)	67 (26.4%)	0.21
Other excisions	7 (0.9%)	11 (4.2%)	0.21	0 (0.00%)	11 (4.3%)	N/A
None	16 (2.2%)	14 (5.4%)	0.17	7 (2.8%)	14 (5.5%)	0.14
Nodal surgery						
SLNB	576 (78.2%)	210 (80.8%)	0.06	196 (77.2%)	205 (80.7%)	0.09
CLND	479 (65.0%)	62 (23.9%)	0.91	164 (64.6%)	60 (23.6%)	0.91
Systemic therapy	292 (39.6%)	155 (59.6%)	0.41	97 (38.2%)	149 (58.7%)	0.42
Radiotherapy	133 (18.1%)	28 (10.8%)	0.21	48 (18.9%)	28 (11.0%)	0.22
Metastasis surgery[c]	≤5 (0.7%)	≤5 (1.9%)	N/A	≤5 (2.0%)	≤5 (2.0%)	N/A

Std. Diff., standardized differences; SD, standard deviation; HNHB, Hamilton Niagara Haldimand Brant; NOS, not otherwise specified; WLE, wide local excision; SLNB, sentinel lymph node biopsy; CLND, completion lymph node dissection.

[a]A standardized difference of ≥0.20 suggests a significant effect size between groups. [b]If more than one type of primary surgery occurred, treatment is prioritized as follows: Amputation > WLE > graft > flap > other excision. [c]Metastasis surgery includes operations for spinal cord, brain, lung, and liver metastases. [d]Categorical responses with ≤5 patients were suppressed due to privacy regulations.

© 2023 Bateni SB et al. *JAMA Dermatology.*

eTable 3. Patient characteristics for unmatched and matched cohorts of Stage IV patients.

	**Unmatched**		**Std. Diff.**[a]	**Matched**		**Std. Diff.**[a]
**Patient Characteristics**	**2007-2012 (N=85)**	**2018-2019 (N=105)**		**2007-2012 (N=56)**	**2018-2019 (N=56)**	
**Age (mean ± SD)**	65.9 ± 17.1	68.01 ± 13.8	0.13	68.5 ± 15.6	70.0 ± 14.4	0.10
**Age (categorized)**						
20-39	7 (8.2%)	≤10 (9.5%)	N/A	≤7 (12.5%)	≤9 (16.1%)	N/A
40-49	12 (14.1%)	≤10 (9.5%)	N/A	≤7 (12.5%)	7 (12.5%)	N/A
50-59	11 (12.9%)	18 (17.5%)	0.12	8 (14.3%)	≤9 (16.1%)	N/A
60-69	12 (14.1%)	29 (27.6%)	0.34	9 (16.1%)	14 (25.0%)	0.22
70-79	23 (27.1%)	24 (22.9%)	0.10	19 (33.9%)	13 (23.2%)	0.24
80+	20 (23.5%)	24 (22.9%)	0.02	13 (23.2%)	17 (30.4%)	0.16
**Sex**						
Female	20 (23.5%)	38 (36.2%)	0.28	12 (21.4%)	26 (46.4%)	0.55
Male	65 (76.5%)	67 (63.8%)	0.28	44 (78.6%)	30 (53.6%)	0.55
**Income quintile**						
1 (Lowest)	16 (18.8%)	21 (20.0%)	0.03	7 (12.5%)	8 (14.3%)	0.05
2	17 (20.0%)	16 (15.2%)	0.13	12 (21.4%)	10 (17.9%)	0.09
3	19 (22.4%)	25 (23.8%)	0.03	13 (23.2%)	14 (25.0%)	0.04
4	16 (18.8%)	22 (21.0%)	0.05	11 (19.6%)	15 (26.8%)	0.17
5 (Highest)	17 (20.0%)	21 (20.0%)	0.00	13 (23.2%)	9 (16.1%)	0.18
**Urban/rural residence**						
Urban	59 (69.4%)	75 (71.4%)	0.04	41 (73.21%)	42 (75.0%)	0.04
Suburban	19 (22.4%)	19 (18.1%)	0.11	≤15 (26.8%)	7 (12.5%)	N/A
Rural	7 (8.2%)	11 (10.5%)	0.08	≤15 (26.8%)	7 (12.5%)	N/A
**Elixhauser comorbidity index (mean ± SD)**	1.04 ± 1.48	0.92 ± 1.67	0.07	1.05 ± 1.39	1.05 ± 1.88	0.00
**Elixhauser comorbidity index (categorized)**						
0-1	58 (68.2%)	81 (77.1%)	0.20	37 (66.1%)	43 (76.8%)	0.24
2-3	19 (22.4%)	16 (15.2%)	0.18	≤19 (33.9%)	7 (12.5%)	N/A
4+	8 (9.4%)	8 (7.6%)	0.06	≤19 (33.9%)	6 (10.7%)	N/A
**Histology (categorized)**						
Melanoma, NOS	29 (34.1%)	68 (64.8%)	0.64	26 (46.4%)	24 (42.9%)	0.07
Nodular melanoma	23 (27.1%)	24 (22.9%)	0.10	14 (25.0%)	21 (37.5%)	0.27
Lentigo maligna/acral lentiginous melanoma	≤17 (20.0%)	≤7 (6.7%)	N/A	≤8 (14.3%)	≤5 (8.9%)	N/A
Superficial spreading melanoma	16 (18.8%)	6 (5.7%)	0.41	8 (14.3%)	6 (10.7%)	0.11
Other	≤17 (20.0%)	≤7 (6.7%)	N/A	≤8 (14.3%)	≤5 (8.9%)	N/A
**Body site (categorized)**						
Head/neck	31 (36.5%)	14 (13.3%)	0.56	18 (32.1%)	10 (17.9%)	0.33
Trunk	29 (34.1%)	30 (28.6%)	0.12	20 (35.7%)	15 (26.8%)	0.19
Arm/shoulder	13 (15.3%)	13 (12.4%)	0.08	8 (14.3%)	10 (17.9%)	0.10
Leg/hip/other	12 (14.1%)	48 (45.7%)	0.74	10 (17.9%)	21 (37.5%)	0.45
**Treatment**						
Primary surgery[b]						
WLE	33 (38.8%)	15 (14.3%)	0.58	18 (32.1%)	10 (17.9%)	0.33
Amputation	≤11 (12.9%)	≤6 (5.7%)	N/A	≤7 (12.5%)	≤5 (8.9%)	N/A
Graft	≤11 (12.9%)	≤6 (5.7%)	N/A	≤7 (12.5%)	≤5 (8.9%)	N/A
Flap	14 (16.5%)	15 (14.3%)	0.06	11 (19.6%)	11 (19.6%)	0.00
Other excisions	13 (15.3%)	10 (9.5%)	0.18	8 (14.3%)	7 (12.5%)	0.05

© 2023 Bateni SB et al. *JAMA Dermatology.*

None	14 (16.5%)	59 (56.2%)	0.91	12 (21.4%)	24 (42.9%)	0.47
Nodal surgery						
SLNB	18 (21.2%)	11 (10.5%)	0.30	10 (17.9%)	6 (10.7%)	0.21
CLND	25 (29.4%)	16 (15.2%)	0.35	15 (26.8%)	7 (12.5%)	0.37
Systemic therapy	32 (37.7%)	80 (76.2%)	0.84	21 (37.5%)	41 (73.2%)	0.77
Radiotherapy	34 (40.0%)	44 (41.9%)	0.04	20 (35.7%)	25 (44.6%)	0.18
Metastasis surgery[c]	≤5 (5.9%)	13 (12.4%)	N/A	≤5 (8.9%)	7 (12.5%)	N/A

Std. Diff., standardized differences; SD, standard deviation; HNHB, Hamilton Niagara Haldimand Brant; NOS, not otherwise specified; WLE, wide local excision; SLNB, sentinel lymph node biopsy; CLND, completion lymph node dissection.

[a]A standardized difference of ≥0.20 suggests a significant effect size between groups. [b]If more than one type of primary surgery occurred, treatment is prioritized as follows: Amputation > WLE > graft > flap > other excision. [c]Metastasis surgery includes operations for spinal cord, brain, lung, and liver metastases. [d]Categorical responses with ≤5 patients were suppressed and place of residence was excluded due to privacy regulations.

© 2023 Bateni SB et al. *JAMA Dermatology.*

eTable 4. Mean healthcare utilization per-person costs in Canadian dollars for the unmatched cohorts stratified by stage.

Type of Service	Cost Description	Stage II		Stage III		Stage IV		All Patients	
		**2007-2012 (N=1,524)**	**2018-2019 (N=421)**	**2007-2012 (N=260)**	**2018-2019 (N=260)**	**2007-2012 (N=85)**	**2018-2019 (N=105)**	**2007-2012 (N=2,346)**	**2018-2019 (N=786)**
Short Episodes (<60 days)	Inpatient hospitalization cost	$12,169	$13,123	$11,971	$13,652	**$18,519**	**$25,678**	**$12,503**	**$16,574**
	Hospital outpatient clinic visit cost	$2,685	$3,103	$4,600	$4,641	**$4,295**	**$5,385**	**$3,367**	**$3,918**
	Same day surgery cost	**$2,378**	**$2,942**	$3,242	$3,314	**$1,771**	**$2,000**	**$2,667**	**$3,008**
	ED visit cost	$762	$777	$836	$908	**$1,031**	**$1,254**	$804	$919
	Dialysis clinic visit cost	$40,319	$90,948	$51,814	$1,178	N/A	$5,247	$44,630	$32,458
	Oncology clinic visit cost	$10,860	$10,304	$20,672	$19,522	**$11,739**	**$17,937**	$15,457	$16,342
	Inpatient rehabilitation cost	$16,986	$22,717	$15,653	$24,412	$25,640	$22,221	$18,494	$22,679
Long-Term Episodes	Complex continuing care cost	$21,252	$27,787	$25,186	$3,302	$11,703	$22,551	$21,213	$17,297
	Long term care cost	$30,976	$31,668	$28,422	$30,749	$20,012	$13,967	$30,311	$29,226
	Inpatient mental health cost	$44,197	$113,730	$40,151	$12,352	N/A	N/A	$42,578	$63,041
Visits/ Claims	FFS GP/FP visit cost	$556	$443	$1,096	$549	$1,018	$1,280	$743	$603
	FFS specialist visit cost	**$3,181**	**$3,856**	**$5,372**	**$4,812**	**$5,719**	**$6,928**	**$3,963**	**$4,583**
	Non-FFS GP/FP visit cost	$18	$15	**$37**	**$17**	$19	$15	$24	$16
	ED-AFA non-FFS visit cost	$200	$223	$213	$274	$165	$255	$203	$247
	Non-FFS medical oncologist visit cost	$687	N/A	$1,308	N/A	$1,291	N/A	$960	N/A
	Radiation oncologist AFA payment apportioned cost	$196	N/A	$228	N/A	$234	N/A	$215	N/A
	Other non-FFS visit cost	$274	$463	$417	$999	$553	$1,591	$347	$818
	Lab cost	$318	$315	$294	$342	$334	$273	$311	$319
	Non-physician cost	$288	$71	$188	$77	$283	$55	$262	$71
	FHO/FHN physician capitation cost	**$224**	**$272**	$203	$227	**$152**	**$186**	$215	$246
	Home care services cost	**$4,034**	**$3,143**	**$4,372**	**$3,246**	**$6,027**	**$3,642**	**$4,272**	**$3,273**
	Non-anticancer related drug cost	$1,593	$1,273	$1,474	$1,582	$1,913	$1,465	$1,569	$1,398

© 2023 Bateni SB et al. *JAMA Dermatology.*

Systemic Therapy	Anticancer related drug cost	**$9,908**	**$40,823**	**$9,343**	**$55,937**	**$8,008**	**$82,421**	**$9,445**	**$61,169**
Total	Total without anticancer drug cost	$22,078	$22,575	**$43,137**	**$37,119**	**$44,749**	**$60,412**	$29,515	$32,440
	Total with anticancer drug cost	$23,014	$27,035	**$46,788**	**$67,669**	**$47,199**	**$122,424**	**$31,359**	**$53,219**

ED, emergency department; FFS, fee for service; GP, general practitioner; FP, family practitioner; AFA, alternative funding arrangement; FHO, family health organization; FHN, family health network. [a]Costs were measured within the first year after melanoma diagnosis. [b]Costs in **bold** indicate a significant effect size between groups based on a standardized difference of ≥0.20.

© 2023 Bateni SB et al. *JAMA Dermatology.*

eTable 5: Mean systemic therapy per-person costs in Canadian dollars for the unmatched cohorts stratified by stage.

Systemic Therapy	Stage II		Stage III		Stage IV		All Patients	
	**2007-2012 (N=1,524)**	**2018-2019 (N=421)**	**2007-2012 (N=260)**	**2018-2019 (N=260)**	**2007-2012 (N=85)**	**2018-2019 (N=105)**	**2007-2012 (N=2,346)**	**2018-2019 (N=786)**
**Chemotherapy**								
Interferon	$9,005	$23,543	$9,411	N/A	$10,127	N/A	$9,286	$23,543
Carboplatin	$735	N/A	$3,603	N/A	$3,475	N/A	$3,261	N/A
Cisplatin	$188	N/A	N/A	N/A	$24	N/A	$106	N/A
Paclitaxel	$187	N/A	$526	N/A	$891	N/A	$710	N/A
Dacarbazine	$2,197	N/A	$2,075	$804	$1,664	N/A	$1,957	$804
Temozolomide	N/A	N/A	$1,160	N/A	$1,653	N/A	$1,407	N/A
**BRAF/MEK inhibitors**								
Dabrafenib	N/A	$6,955	N/A	$10,130	N/A	$19,627	N/A	$12,691
Trametinib	N/A	$8,482	N/A	$10,173	N/A	$19,456	N/A	$12,884
Vemurafenib	$8,450	N/A	$6,186	$822	$23,395	N/A	$10,262	$822
**Checkpoint inhibitors**								
Ipilimumab	$92,800	$79,253	$92,800	$81,618	$55,796	$84,826	$83,549	$83,345
Nivolumab	N/A	$34,132	N/A	$54,136	N/A	$29,244	N/A	$44,431
Pembrolizumab	N/A	$37,345	N/A	$63,267	N/A	$63,687	N/A	$60,316
**All systemic therapies**	$9,908	$40,823	$9,343	$55,937	$8,008	$82,421	$9,445	$61,169

[a]Costs were measured within the first year after melanoma diagnosis.

© 2023 Bateni SB et al. *JAMA Dermatology.*

eTable 6: Mean systemic therapy per-person costs in Canadian dollars for the matched cohorts stratified by stage.

Systemic Therapy	Stage II		Stage III		Stage IV		All Patients	
	**2007-2012 (N=421)**	**2018-2019 (N=421)**	**2007-2012 (N=254)**	**2018-2019 (N=254)**	**2007-2012 (N=56)**	**2018-2019 (N=56)**	**2007-2012 (N=731)**	**2018-2019 (N=731)**
**Chemotherapy**								
Interferon	$9,424	$23,543	$9,491	N/A	$9,759	N/A	$9,475	$23,543
Carboplatin	N/A	N/A	$5,123	N/A	$3,839	N/A	$4,206	N/A
Cisplatin	$188	N/A	N/A	N/A	$24	N/A	$106	N/A
Paclitaxel	N/A	N/A	$754	N/A	$964	N/A	$894	N/A
Dacarbazine	$1,961	N/A	$2,198	$804	$1,759	N/A	$1,953	$804
Temozolomide	N/A	N/A	$1,126	N/A	$2,273	N/A	$1,699	N/A
**BRAF/MEK inhibitors**								
Dabrafenib	N/A	$6,955	N/A	$8,114	N/A	$29,002	N/A	$11,937
Trametinib	N/A	$8,482	N/A	$8,370	N/A	$26,737	N/A	$12,071
Vemurafenib	$8,450	N/A	$8,615	$822	$23,395	N/A	$14,494	$822
**Checkpoint inhibitors**								
Ipilimumab	$69,600	$79,253	$92,800	$81,618	$55,796	$81,659	$72,732	$81,064
Nivolumab	N/A	$34,132	N/A	$54,540	N/A	$27,151	N/A	$46,779
Pembrolizumab	N/A	$37,345	N/A	$63,267	N/A	$67,548	N/A	$60,727
**All systemic therapies**	$10,309	$40,823	$9,764	$55,699	$9,318	$79,358	$9,860	$56,874

[a]Costs were measured within the first year after melanoma diagnosis.

© 2023 Bateni SB et al. *JAMA Dermatology.*

eTable 7. Time toxicity sensitivity analysis in days including virtual visits and home care visits.

Stage	Time Toxicity	2007-2012<br>Mean ± SD	2018-2019<br>Mean ± SD	Std. Diff.
II	Original	31.90 ± 28.21	31.36 ± 30.43	0.02
	Original plus virtual/home visits	45.96 ± 45.16	48.38 ± 51.22	0.05
III	Original	47.93 ± 32.80	42.59 ± 24.04	0.19
	Original plus virtual/home visits	73.43 ± 48.69	62.72 ± 44.04	0.23
IV	Original	44.18 ± 26.47	58.67 ± 43.78	0.40
	Original plus virtual/home visits	73.66 ± 61.45	99.84 ± 78.75	0.37

© 2023 Bateni SB et al. *JAMA Dermatology.*
