# Supplementary material for: Changes in Health Care Costs, Survival, and Time Toxicity in the Era of Immunotherapy and Targeted Systemic Therapy for Melanoma
Source: JAMA Dermatol. 2023 Sep 6;159(11):1195–204. doi: 10.1001/jamadermatol.2023.3179 (PMC10483386; doi:10.1001/jamadermatol.2023.3179)
Supplement: Supplement 2. — Data Sharing Statement [file jamadermatol-e233179-s002.pdf]

Data Sharing Statement

Bateni. Changes in Health Care Costs, Survival, and Time Toxicity in the Era of Immunotherapy and Targeted Systemic Therapy for Melanoma. *JAMA Dermatol*. Published September 06, 2023. doi:10.1001/jamadermatol.2023.3179

Data
Data available: No

Additional Information
Explanation for why data not available: Institutional and provincial privacy regulations on the use of administrative data.
